# Batesian Mimicry Converges toward Inaccuracy in Myrmecomorphic Spiders

**DOI:** 10.1093/sysbio/syaf037

**Published:** 2025-05-19

**Authors:** Michael B J Kelly, Shahan Derkarabetian, Donald James McLean, Ryan Shofner, Cristian J Grismado, Charles R Haddad, Gerasimos Cassis, Gonzalo Giribet, Marie E Herberstein, Jonas O Wolff

**Affiliations:** School of Natural Sciences, Macquarie University, 4–6 Eastern Road, Macquarie Park, NSW 2109, Australia; Evolutionary Biomechanics, Zoological Institute and Museum, University of Greifswald, Loitzer Strasse 26, 17489 Greifswald, Germany; Museum of Comparative Zoology, Department of Organismic and Evolutionary Biology, Harvard University, 26 Oxford Street, Cambridge, MA 02138, USA; School of Natural Sciences, Macquarie University, 4–6 Eastern Road, Macquarie Park, NSW 2109, Australia; Evolution & Ecology Research Centre, School of Biological, Earth and Environmental Sciences, University of New South Wales, Gate 11 Botany Street, Randwick, NSW 2052, Australia; Museo Argentino de Ciencias Naturales “Bernardino Rivadavia”, Consejo Nacional de Investigaciones Científicas y Técnicas (CONICET), Avenida Ángel Gallardo 470, C1405DJR Buenos Aires, Argentina; Department of Zoology and Entomology, University of the Free State, 205 Nelson Mandela Drive, Bloemfontein 9300, Republic of South Africa; Evolution & Ecology Research Centre, School of Biological, Earth and Environmental Sciences, University of New South Wales, Gate 11 Botany Street, Randwick, NSW 2052, Australia; Museum of Comparative Zoology, Department of Organismic and Evolutionary Biology, Harvard University, 26 Oxford Street, Cambridge, MA 02138, USA; School of Natural Sciences, Macquarie University, 4–6 Eastern Road, Macquarie Park, NSW 2109, Australia; Leibniz Institute for the Analysis of Biodiversity Change, Adenauerallee 160, 53113 Bonn, Germany; Institute für Cell- und Systems Biology of Animals, University of Hamburg, Martin-Luther-King Platz 3, 20146 Hamburg, Germany; School of Natural Sciences, Macquarie University, 4–6 Eastern Road, Macquarie Park, NSW 2109, Australia; Evolutionary Biomechanics, Zoological Institute and Museum, University of Greifswald, Loitzer Strasse 26, 17489 Greifswald, Germany

**Keywords:** Araneae, Castianeirinae, Corinnidae, macroevolution, Myrmarachnini, Salticidae, trait evolution

## Abstract

Batesian mimicry is an impressive example of convergent evolution driven by predation. However, the observation that many mimics only superficially resemble their models despite strong selective pressures is an apparent paradox. Here, we tested the “*perfecting hypothesis*”, that posits that inaccurate mimicry may represent a transitional stage at the macroevolutionary scale by performing the hereto largest phylogenetic analysis (in terms of the number of taxa and genetic data) of ant-mimicking spiders across two speciose but independent clades, the jumping spider tribe Myrmarachnini (Salticidae) and the sac spider subfamily Castianeirinae (Corinnidae). We found that accurate ant mimicry evolved in a gradual process in both clades, by an integration of compound traits contributing to the ant-like habitus with each trait evolving at different speeds. Accurate states were highly unstable at the macroevolutionary scale likely because strong expression of some of these traits comes with high fitness costs. Instead, the inferred global optimum of mimicry expression was at an inaccurate state. This result reverses the onus of explanation from inaccurate mimicry to explaining the exceptional evolution and maintenance of accurate mimicry and highlights that the evolution of Batesian mimicry is ruled by multiple conflicting selective pressures.

Mimicry, a paradigm of convergent evolution (Reed et al. 2011), is often noted as evidence of the power of natural selection in generating spectacular adaptations (Johnstone 2002; Kazemi et al. 2014). In Batesian mimicry (mimicry by deceptive appearance), it should be expected that those mimics that evolve traits more closely resembling the traits of their model will gain the greater fitness benefit as the ability of predators to discriminate between the mimic and its dangerous or unpalatable model is reduced (i.e., Batesian mimicry theory; Ruxton et al. 2019). There is evidence that traits involved in mimicry are under strong selective pressure to better resemble those characteristics seen in their respective model (e.g., Ceccarelli 2013), suggesting that natural selection is driving the ever-increasing perfection in mimic accuracy (Mappes and Alatalo 1997; Wickler 2013; Taylor et al. 2016).

However, contrary to theory (e.g., Mappes and Alatalo 1997), mimicry does not consistently involve the convergence of the full range of model traits, resulting in widespread cases of imperfect or inaccurate mimicry (Sherratt and Peet-Paré 2017). For example, many species of hoverflies are poor mimics of wasps and bees (Edmunds 2000), many species of nonvenomous king snakes imprecisely mimic deadly coral snakes (Kikuchi and Pfennig 2010), and many myrmecomorphic (i.e., ant-mimicking) spiders inaccurately resemble their ant model (Cushing 2012). Several nonmutually exclusive hypotheses have been proposed to explain the prevalence and maintenance of inaccurate mimics (reviewed in McLean et al. 2019). Most of these hypotheses assume that inaccurate mimics are at an evolutionary stable state and that further refinement would not provide any additional fitness benefit or may even reduce the mimic’s fitness. Alternatively, inaccurate mimics may be under continuing directional selection and represent a transitional stage in an evolutionary trajectory toward a phenotypic adaptive optimum (discussed in Fisher 1930; Nur 1970; Edmunds 2000; Gilbert 2005; McLean et al. 2019).

In the “two-step” theory of mimetic evolution (Poulton 1912), a large mutational change results in a relatively close resemblance to the model (potentially bypassing the inaccurate mimetic phenotype), with subsequent refinement by smaller mutations at several loci (Allen and Cooper 1995). Alternatively, accurate mimics may evolve through incremental trait evolution, requiring many mutational or recombination steps (Fisher 1930), building on Darwin’s concept of gradualism (Darwin 1859). This idea has been termed the evolutionary lag or *perfecting* hypothesis (sensu McLean et al. 2019), with limited empirical studies that have found mixed results: support in ant-mimic spiders (McIver and Stonedahl 1993; Pekár 2014) and coral snake mimicry (Kikuchi and Pfennig 2010) but not in wasp-mimicking hoverflies (Holloway et al. 2002). Irrespective of the precise mechanism of transition (gradual or punctuated), testing the idea of perfecting in mimicry requires a phylogenetic approach to determine whether there are observable trends in mimic accuracy (see Pennell et al. 2014 and citations within).

Here, we test the perfecting hypothesis using myrmecomorphic spiders (Cushing 1997, 2012) who gain protection from predators by mimicking unpalatable or aggressive ants (Cushing 1997). In spiders, myrmecomorphy has evolved in at least 16 families and 85 genera (Reiskind 1971; McIver and Stonedahl 1993; Cushing 1997, 2012; Pekár 2014). Although spiders are generally palatable and are readily preyed upon by numerous predators (Edmunds 1974), myrmecomorphy is effective against visually guided predators including insectivorous birds (Moya-Laraño et al. 2013), praying mantises (Nelson et al. 2006; Ramesh et al. 2016), wasps (Uma et al. 2013), and various araneophagous spiders (Durkee et al. 2011; Huang et al. 2011; Nelson 2012).

Accurate myrmecomorphy is a remarkable modification of the typically highly stable body shape of spiders (Wolff et al. 2021, 2022): from a stocky arachnid with two tagmata and eight legs into a thin ant-like habitus creating the illusion of three tagmata separated by narrow constrictions, and with two antennae and six legs (Shamble et al. 2017). Traits such as the elongation of the body, extension of the pedicel (the narrow stalk connecting the cephalothorax and the abdomen; Foelix 2011), constrictions to the body, color changes, patches of hair-like seta, and thinning of all legs are integrated to produce a convincing ant-like illusion (Kelly et al. 2021). Due to the number and extent of these morphological changes, we assume that the evolution of accurate myrmecomorphy occurs gradually in spiders, and that observed cases of inaccurate myrmecomorphy may represent unstable intermediate stages toward mimicry perfection.

To test this, we conducted the first comprehensive macroevolutionary study of myrmecomorphy in spiders across two separate clades that have evolved myrmecomorphy independently (Pekár 2014)—the jumping spider tribe Myrmarachnini (Salticidae) and the sac spider subfamily Castianeirinae (Corinnidae). Both clades profoundly differ in their ecology and basic body plan, offering an opportunity to test whether the evolution of Batesian mimicry follows common rules of convergent evolution independent of evolutionary history and niche adaptation. We used target-enrichment sequencing of ultraconserved elements (UCEs; Faircloth et al. 2012), a method of genome subsampling that is phylogenetically informative at both shallow- and deep-level timescales (e.g., Hedin et al. 2019, 2020; Kulkarni et al. 2020; Xu et al. 2021; also see Gustafson et al. 2020 and citations within). As many of the ant models that are mimicked by these spiders are unknown and not all our specimens have been identified to species level, we generated a matrix of myrmecomorphic traits based on a comprehensive literature analysis (Kelly et al. 2021) and used it to analyze trait distribution across the fossil-calibrated phylogenies. Support for the perfecting hypothesis was given if moderately accurate species were nested in clades of inaccurate species, and if accurate species were nested in clades of moderately accurate species. In contrast, the perfecting hypothesis was not supported if highly accurate species were nested in clades of nonmimic or inaccurate species. To test whether selection acts preferentially toward inaccurate mimicry, we fitted different evolutionary models with and without the *a priori* assumption of global adaptive peaks. We hypothesized that inaccurate states represent unstable, transitional stages and that adaptative peaks are found at highly accurate mimicry (“perfecting” hypothesis).

## Materials and Methods

### Taxon Sampling

In total, 204 specimens of Myrmarachnini (Salticidae) and 91 specimens of Castianeirinae (Corinnidae) were included in our study. The specimens were obtained from our own fieldwork (*n* = 59), from university and research institute collections (*n* = 171), and from museum collections (*n* = 90). To cover a large proportion of the extant phenotypic diversity of myrmecomorphic spiders (specifically in Myrmarachnini) we included currently undescribed species and specimens for which the identification was difficult due to the incomplete taxonomy of the group. For notes on species identification, see online Appendix 1 (I. Species Identification). Additionally, a single outgroup species with published UCE sequence data from Azevedo et al. (2022) was included. Sampling from our own fieldwork involved the visual inspection of tree trunks (including underneath bark), branches, foliage, vegetation, leaf litter, under stones, as well as the use of beating trays. Although we attempted a broad global coverage in our species sampling, we recognize that some geographic regions were underrepresented, such as South American Castianeirinae. Outgroups for Myrmarachnini included members of the subfamilies Hisponinae (*n* = 4) and Salticinae (*n* = 12; Maddison 2015). Outgroups for Castianeirinae consisted of representatives of the subfamily Corinninae (*n* = 10; Wheeler et al. 2017). All specimen data, including collection information, are listed in online Appendix 2 (Supplementary Data Set S1—Sample Information).

### Trait Selection and Evaluation

Specimens were filmed (alive) using a Basler Ace 640 × 480 pixel USB 3.0 high-speed video camera (Basler AG, Ahrensburg, Germany) or photographed (preserved) using either a VHX-6000 digital microscope (Keyence Corporation, Osaka), a Cannon EOS 80D camera mounted onto a Motic SMZ-171 microscope, or alternatively using a Leica DFC 290 digital camera mounted on a Leica M165 C stereoscopic microscope with the focal planes aligned with Helicon Focus 4.62.2. These images were then used for biometrical measurements (in millimeters) using ImageJ v1.51 (Schneider et al. 2012). Some specimens obtained from tissue collections were not available for measurements and some specimens were damaged or had a distorted morphology. In these few instances, biometric measurements were made on figures published in the taxonomic literature (e.g., Davies and Żabka 1989; Raven 2015).

Appropriate quantification of accurate mimicry is often noted as a challenging step in studies focused on mimetic variation (Penney et al. 2012; Kelly et al. 2021). The ultimate method is often intuitively considered to be via experimentation using relevant predator assessment trials or alternatively through the direct assessment of similarity between the mimic’s phenotype and that of its’ putative model (i.e., geometric morphometrics or linear morphometrics; see Kelly et al. [2021] for a discussion on methodological approaches for mimic assessment). However, these approaches of quantification only become feasible when considering a small number of identified mimics, a knowledge of the relevant biological predators, and/or when the putative models have been identified. In studies that include a large sampling of mimic species (which is largely based on museum specimens with limited natural history data available) across a global distribution, such as the present study, these methodologies of predator trials and direct mimic/model comparison would be an unviable task as many predators and models have not been empirically validated for many mimic species, including those used here.

**Figure 1.**
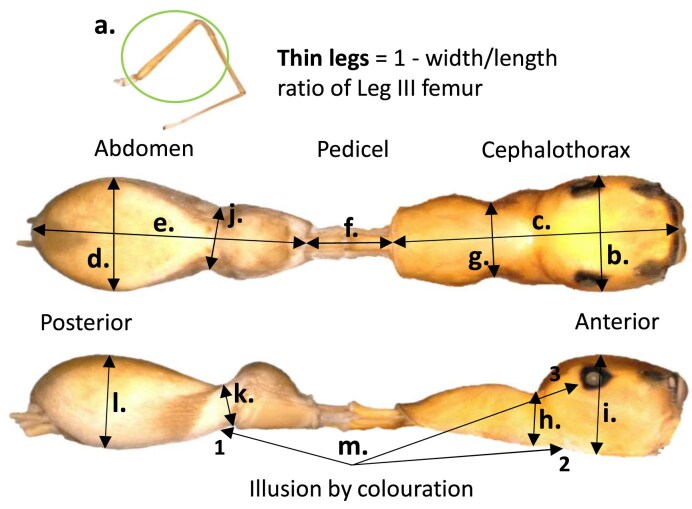
Traits quantified to determine myrmecomorphy, after Kelly et al. (2021): 1) thin legs = 1–width leg III femur/length leg III femur (a); 2) elongation of cephalothorax = 1–cephalothorax width (b)/cephalothorax length (c); 3) elongation of abdomen = 1–abdomen width (d)/abdomen length (e); 4) elongation of pedicel = pedicel length (f)/total body length (c + e + f); 5) constriction of the cephalothorax (in dorsal view) = 1–cephalothorax width at point of constriction (g)/cephalothorax width at widest point (b); 6) constriction of the cephalothorax (in lateral view) = 1–cephalothorax height at point of constriction (h)/cephalothorax height at highest point (i); 7) constriction of the abdomen (in dorsal view) = 1–abdomen width at point of constriction (j)/abdomen width at widest point (d); 8) constriction of the abdomen (in lateral view) = 1–abdomen height at point of constriction (k)/abdomen height at highest point (l); 9) illusion by coloration (m) = scored based on the presence of the illusion by coloration traits 1–3 (i.e., single trait = 0.334, two traits = 0.667, and all three traits = 1.0), 1 = transverse band or stripe of lightly colored setae on the abdomen creating the illusion of a separation into a petiole (or postpetiole) and abdomen, 2 = transverse band or stripe of lightly colored setae on the cephalothorax creating the illusion of a separation into a head and thorax, and 3 = darkening of the area surrounding the posterior lateral eye creating the illusion of only two large compound eyes. Overall mimic accuracy = average of all trait scores combined (i.e., all traits summed and divided by 9).

Here, we have elected to implement a trait-based assessment of mimetic accuracy following Kelly et al. (2021) that utilizes several mimetic traits repeatedly noted in the literature as resulting in an overall ant-like morphology in spiders (e.g., Cloudsley-Thompson 1995; Cushing 1997, 2012; Deeleman-Reinhold 2001; Edmunds 2006; Durkee et al. 2011; see Fig. 1 and Supplementary Table S1 for selected myrmecomorphic traits and method of trait and overall mimetic accuracy quantification). We acknowledge that this approach is largely based on human assessment, albeit expert opinion, of those traits considered important in contributing to ant-like morphology in myrmecomorphic spiders. Additionally, we recognize that ants possess diverse morphologies and behaviors and that other phenotypic aspects may also be important cues for predator recognition, such as other morphological, behavioral, and olfactory traits.

Further, we acknowledge that predator vision will deviate from human perception; however, we assume that these selected traits are likely salient to potential predators and are under selective pressure that has resulted in the extreme morphological adaptations observed in myrmecomorphic species. For example, previous experiments using birds (Dittrich et al. 1993) and spiders (Nelson 2012) showed that predators may use comparable cues as humans for assessments of mimetic accuracy.

Finally, based on a former comparison of methods (Kelly et al. 2021), the use of trait-based assessment should result in a meaningful description of mimetic accuracy as it has been shown to be highly correlated with other quantification methods (e.g., geometric morphometrics and linear morphometrics) whereas remaining feasible for the use of large-scale museum specimens. This approach allows each selected trait to be calculated as a (proportional) continuous accuracy score that can be viewed as an expression of the trait’s state. Mimicry accuracy in this study was defined as follows: accuracy score <0.15 (nonmimic); 0.15 to <0.30 (inaccurate mimic); and ≥0.30 (accurate mimic). Among the inaccurate mimics, we further distinguished between: 1) low levels of mimicry accuracy (scores 0.15–0.19) and 2) moderate levels of mimicry accuracy (scores 0.20–0.29).

### DNA Extraction and UCE Library Preparation

Genomic DNA was extracted from either leg(s) or whole individuals and performed using two different approaches: 1) DNeasy Blood and Tissue Kit (Qiagen, Valencia, CA) following the manufacturer’s protocol and 2) an alternative extraction protocol adapted from Tin et al. (2014) and Derkarabetian et al. (2019), used for 12 samples containing degraded DNA. In brief, the adapted Tin et al. (2014) and Derkarabetian (2019) protocols involved washing samples in Molecular Biology Grade Water (Mediatech, Inc.) to remove any ethanol from the storage process, then drying and placing samples into tubes with 200 µL of extraction buffer. Samples were then incubated in a water bath at 58 °C for 24 h for tissue lysis to occur. Following tissue lysis, bead purification using Serapure SPRI beads (Rohland and Reich 2012; Glenn et al. 2019) was performed to separate the Serapure SPRI beads attached to the genomic and mitochondrial DNA from proteins and other contaminants. Extracted DNA was quantified using both high-sensitivity and broad-range assays on a Qubit fluorometer (Life Technologies, Inc.). DNA sample details are listed in online Appendix 2 (Supplementary Data Set S1—Sample Information).

Following this, gel electrophoresis was conducted on a 1% agarose gel to view DNA distribution and assess quality to determine the appropriate amount of sonication time. Sonication of 500 ng of DNA, in a sample volume of 130 µL of AE buffer, was performed to fragment samples collected in the field and/or stored in 95% ethanol using a Covaris S220 sonicator for 80 s with a peak incidence power of 105.0, duty factor of 5%, and 200 cycles per burst. From the resulting fragmented DNA, 4 µL were run out on a 1.2% agarose gel to confirm sonication success and view the distribution of DNA fragments. The target size range for the sonicated DNA fragments was between 300 and 500 bp, but not exceeding 1000 bp. The remaining samples, where potential partial DNA degradation was expected (i.e., preserved specimens stored in <95% ethanol and museum specimens), were not sonicated. A maximum starting quantity of 500 ng total DNA was used for UCE library preparation. UCE library preparation followed the protocol of Starrett et al. (2017) and the UCE website (https://www.ultraconserved.org/). Pooled libraries then underwent target enrichment at 60 ºC for 24 h using the MYbaits Arachnida 1.1K version 1 kit (Arbor Biosciences, Ann Arbor, MI; Faircloth 2017), which targets ∼1120 UCE loci (Faircloth 2017; Starrett et al. 2017) following the protocol detailed in the Hybridization Capture for Targeted NGS manual v4.01 (https://arborbiosci.com/mybaits-manual/). Note that subsequent to the processing of our specimens in 2018/2019 an Araneae probe set (Kulkarni et al. 2020) and an RTA clade probe set (Zhang et al. 2023) were developed, therefore these options were not available to us at the time. However, the Arachnid probe set has been successfully used in a variety of arachnid clades, even at the level of population, and it is a highly versatile probe set (e.g., Derkarabetian et al. 2022; Heine et al. 2024). Libraries were then sequenced on a NovaSeq 6000 at the Bauer Core Facility at Harvard University with 150 bp paired-end reads.

### Sequence Data Processing

Due to the large number of reads, duplicate reads were removed prior to quality control using CD-HIT-DUP contained within the CD-HIT package (Li and Godzik 2006; Fu et al. 2012). Processing of raw demultiplexed read data was performed using the PHYLUCE v1.6.8 pipeline (available at https://phyluce.readthedocs.io/en/latest/; Faircloth 2016). The Trimmomatic wrapper (Bolger et al. 2014), Illumiprocessor (Faircloth 2013), was implemented using default settings to remove adapters and perform quality-control trimming. Clean reads were then assembled into contigs using Trinity v2.1.1 (Grabherr et al. 2011) with default settings and ABySS v1.5.2 (using 61-kmer value setting; Simpson et al. 2009), and the results were combined into a single assembly file, thereby increasing the overall number of UCE loci per sample relative to using a single assembly method (e.g., Hedin et al. 2019). Probes were matched to contigs using minimum coverage and minimum identity values of 65. The UCE loci were aligned using MAFFT (Katoh and Standley 2013) and trimmed using Gblocks (Castresana 2000; Talavera and Castresana 2007), with custom blocks settings (*b*1 = 0.5, *b*2 = 0.5, *b*3 = 6, and *b*4 = 6) applied in the PHYLUCE pipeline. Two separate data sets were created, one containing the Myrmarachnini tribe (including outgroup species), the other containing the Castianeirinae subfamily (including outgroup species). We chose different gene occupancy thresholds across data sets to maximize the number of loci without drastically increasing computational time, whereas also accounting for the fact that the data sets include different numbers of samples and different levels of divergence. For each of the data sets, a matrix was created: Myrmarachnini = 75% occupancy matrix (i.e., including loci captured for 0.75 proportion of taxa); and Castianeirinae = 50% occupancy matrix (i.e., loci captured for 0.50 proportion of taxa). UCE alignments were then imported into Geneious 11.1.5 (http://www.geneious.com; Kearse et al. 2012) and visually inspected to remove any nonhomologous sequences and obvious alignment errors.

### Phylogenetic Analyses

Phylogenetic inference based on maximum likelihood (ML) was performed in IQ-TREE v2.1.3 (Minh et al. 2020). Each UCE locus was treated as a separate partition; partitions were set with linked branch lengths and independent evolutionary rates. Best-fitted models of molecular evolution for each partition were determined by ModelFinder (Kalyaanamoorthy et al. 2017), as implemented in IQ-TREE. Branch support was estimated with an ultrafast bootstrap approximation, using 1000 replicates (Hoang et al. 2018).

For the Bayesian phylogenetic inference (BI), all UCE loci were concatenated into a single partition. Following tests, to enhance computability, individual loci within each data set were reprocessed with more conservative settings (*b*1 = 0.5, *b*2 = 0.85, *b*3 = 4, and *b*4 = 8) using Gblocks v0.91b (Castresana 2000; Talavera and Castresana 2007), which removed the most variable distal flanking regions of the sequences. The BI analysis was performed using BEAST 2 v2.6.6 (Bouckaert et al. 2019), using the following priors: HKY + gamma site model, a log-normal relaxed molecular clock, random starting tree, and the birth–death evolutionary model. To obtain divergence time estimates, monophyly constraints were set based on published phylogenomic information (Wheeler et al. 2017; Fernández et al. 2018), with the age prior of the common ancestor informed by fossil taxonomy or secondary calibration points from published analyses (Magalhães et al. 2020). For Myrmarachnini, the following (primary) calibration points were used following Bodner and Maddison (2012) with a uniform distribution: 1) the root of the tree representing the age of the most recent common ancestor of Salticidae (minimum 44 Ma based on the oldest fossil salticid, Baltic Amber; maximum 100 Ma based on absence in Cretaceous amber) and 2) at Hisponinae spp. (*Hispo* and *Tomocyrba*), representing the age of the most recent common ancestor of Salticoida (minimum 16 Ma based on oldest fossil Salticoida, Dominican Amber; maximum 49 Ma based on absence in Baltic Amber). For Castianeirinae, we used a single secondary calibration point following Magalhães et al. (2020), with a normal distribution between *Falconina* and *Nyssus*, previously inferred node age 15–55 Ma. The proportion of invariant sites for Castianeirinae was 0.6387, with *Austrophaea zebra* set as the outgroup. For Myrmarachnini, the proportion of invariant sites was 0.6032, with members of the subfamily Hisponinae and Salticinae set as the outgroup.

Three Markov chain Monte Carlo (MCMC) simulations were run for each taxonomic group. Convergence of the models was checked by examining the BEAST logs in Tracer v1.7.2 (Rambaut et al. 2018) to visualize parameter evolution and determine the effective sample size (ESS) of parameters. For the corinnid data set, sampling over 200 million generations was sufficient to reach convergence and obtain a good ESS; for the (larger) salticid data set, 400 million generations were adequate.

The inferred trees were visualized using FigTree v1.4.3 (Rambaut 2016) and a time-stamped ultrametric consensus tree was calculated from a posterior sample from each of three independent MCMC runs using *TreeAnnotator* from the BEAST package.

### Comparative Analyses

To infer local trait evolution, ancestral character estimation was performed using the *ace* function in *ape* (Paradis and Schliep 2019). Plotting of the extant and estimated ancestral trait states was done using *phytool ’s contMap* function. Note that this method by default uses node state estimated based on the Brownian motion (BM, unconstrained trait diffusion; Felsenstein 1973) model. As mimicry trait evolution was predicted to deviate from BM, its fit was compared against Ornstein–Uhlenbeck (OU, model assumes evolution is stabilized around an optimal trait value, an extension of the BM model; Butler and King 2004) and early burst (EB, BM with evolutionary rate deceleration; Harmon et al. 2010) models, using the *fitContinuous* function and corrected Akaike information criterion weights (AICcWs) in *geiger*. If OU was the best-fitted model, the node states were calculated by rerooting the alpha-transformed tree (using the alpha from the fitted OU model) in each node. If EB was the best-fitted model, the same was done but scaling the tree by the parameter *a* of the fitted EB model. The calculated node states were then fixed when creating the *contMap*.

To infer the evolutionary trait optimum of mimicry accuracy, we further analyzed the parameter estimates of the best-fitted OU models, that is, *σ*² (the macroevolutionary rate of trait change), *θ* (the global macroevolutionary trait optimum, or root state), and *α* (the “pull-factor” indicating the strength of selection toward the trait optimum *θ*). Due to conflicting selective pressures, mimicry accuracy could also evolve toward disparate global optima; to test this, we fitted the Fokker–Planck–Kolmogorov (FPK) model by Boucher et al. (2018) with the *BBMV* package, and plotted the polynomial function defined by the inferred parameters *a, b*, and *c*, indicating the global adaptive landscape of mimicry evolution.

The mimicry accuracy score used in this study collapses nine different traits contributing to myrmecomorphy into a single variable. To enhance the understanding of how the included traits differ in their evolutionary dynamics, we performed separate analyses for each trait using the same framework as described for the mimicry accuracy score. The ninth trait “Illusion by coloration” was excluded here as it is a quasicategorical trait. Also, note that in Castianeirinae, the traits “Dorsal cephalothorax constriction,” “Dorsal abdominal constriction,” and “Lateral abdominal constriction” were expressed only in a very limited number of specimens, so the fit of evolutionary models to such pointed trait variations should be interpreted with care.

To further explore the phylogenetic strength of the traits, we calculated the phylogenetic signal using Pagel’s lambda and Blomberg’s *K*. Calculations were done using the *phylosig* function of *phytools* (Revell 2012). To test the effect of phylogenetic uncertainty on the results (i.e., topology and branch lengths due to uncertainty in divergence time estimation), for each clade all phylogenetic comparative analyses were repeated across a sample of 100 trees randomly drawn from the combined posterior of all MCMCs of the Bayesian tree inference (after removal of the 30% burn-in). Data were summarized and visualized using the *sensiPhy* package (Paterno et al. 2018), based on the script in Wolff et al. (2019). To investigate the phylogenetic sensitivity of the inferred evolutionary model fit and parameter estimates, we ran the comparative analysis explained above across a sample of one hundred trees randomly drawn from the combined MCMCs of the Bayesian tree inference (excluding burn-in).

## Results

### Trait Variation

We used a numeric index of myrmecomorphy representing the degree of modification of the basic body shape of modern spiders across a set of nine traits (Kelly et al. 2021). In Castianeirinae (*n* = 91), the mimic accuracy was relatively low (average = 0.18, min–max: 0.10–0.30, SD± 0.04) with 21 specimens scored as nonmimetic, 69 scored as an inaccurate mimic (38 low, 31 moderate accuracy), and only 1 species considered to be an accurate mimic, *Myrmecium bifasciatum*. In Myrmarachnini (*n* = 204), overall mimic accuracy was higher (average = 0.31, min–max: 0.16–0.47, SD± 0.07), with no specimens scored as nonmimetic, 77 scored as inaccurate (9 low, 68 moderate accuracy), and 127 specimens scored as accurate mimics.

An important myrmecomorphic trait in Myrmarachnini is the prevalence of dorsal and lateral constrictions to both the abdomen and the cephalothorax relative to Castianeirinae: Myrmarachnini—*n* = 198 out of 204 with constriction; Castianeirinae—*n* = 15 out of 91 with constriction. The degree and prevalence of traits including the elongation of the cephalothorax, the abdomen, and the pedicel were both higher in Myrmarachnini, contributing to their higher overall mimic accuracy indices (Table 1). Illusion by coloration was more consistent within Myrmarachnini (*n* = 165) than Castianeirinae (*n* = 27). For all biometric measurements, see online Appendix 3 (Supplementary Data Set S2—Biometric Measurements) .

**Table 1.** The minimum, maximum, mean, and sample standard deviation (SD**±**) values for the eight selected morphometric traits, and the overall mimic accuracy scores (shown in bold) for Castianeirinae and Myrmarachnini.

Characteristic	Minimum	Maximum	Mean	SD (±)
**Castianeirinae (*n* = 91)**				
Thin legs	0.59	0.89	0.71	0.06
Elongation of cephalothorax	0.17	0.66	0.33	0.09
Elongation of abdomen	0.12	0.61	0.39	0.11
Elongation of pedicel	0.00	0.11	0.03	0.02
Lateral cephalothorax constriction	0.00	0.39	0.01	0.05
Dorsal cephalothorax constriction	0.00	0.63	0.01	0.07
Lateral abdominal constriction	0.00	0.18	0.01	0.03
Dorsal abdominal constriction	0.00	0.22	0.01	0.03
**Overall mimic accuracy**	**0.10**	**0.30**	**0.18**	**0.04**
**Myrmarachnini (*n* = 204)**				
Thin legs	0.59	0.84	0.75	0.04
Elongation of cephalothorax	0.22	0.66	0.47	0.06
Elongation of abdomen	0.20	0.80	0.50	0.12
Elongation of pedicel	0.00	0.24	0.07	0.05
Lateral cephalothorax constriction	0.00	0.48	0.19	0.10
Dorsal cephalothorax constriction	0.00	0.44	0.18	0.10
Lateral abdominal constriction	0.00	0.48	0.16	0.13
Dorsal abdominal constriction	0.00	0.39	0.12	0.13
**Overall mimic accuracy**	**0.16**	**0.47**	**0.31**	**0.07**

Source: Kelly et al. 2021 and Supplementary Table S1 for definitions and method of measurement

### Sequencing Results (UCEs)

Specimen sequence data can be found in online Appendix 2 (Supplementary Data Set S1—Sample Information). For information on availability of UCE sequence data see Data Availability section. For the Castianeirinae data set, the number of reads pass QC ranged between 145,707 and 19,183,248 (mean = 6,955,279; SD± 4,756,270), the number of contigs ranged between 10,855 and 1,853,713 (mean = 583,502; SD± 419,282), and the number of UCE loci (50% occupancy matrix) ranged between 15 and 809 (mean = 636; SD± 258). For the Myrmarachnini data set, the number of reads pass QC ranged between 209,873 and 37,817,349 (mean = 5,780,188; SD± 3,973,659), the number of contigs ranged between 9924 and 5,562,935 (mean = 550,682; SD± 475,362), and the number of UCE loci (75% occupancy matrix) ranged between 24 and 603 (mean = 542; SD± 120).

### Phylogenetic Analyses

ML analysis of both Castianeirinae (Supplementary Fig. S1) and Myrmarachnini (Supplementary Fig. S2) UCE data sets produced a phylogeny with the vast majority of the ultrafast bootstrap support (UBS) values ≥ 95%. The lower supported nodes were mostly associated with historical museum specimens with highly fragmented DNA.

**Figure 2.**
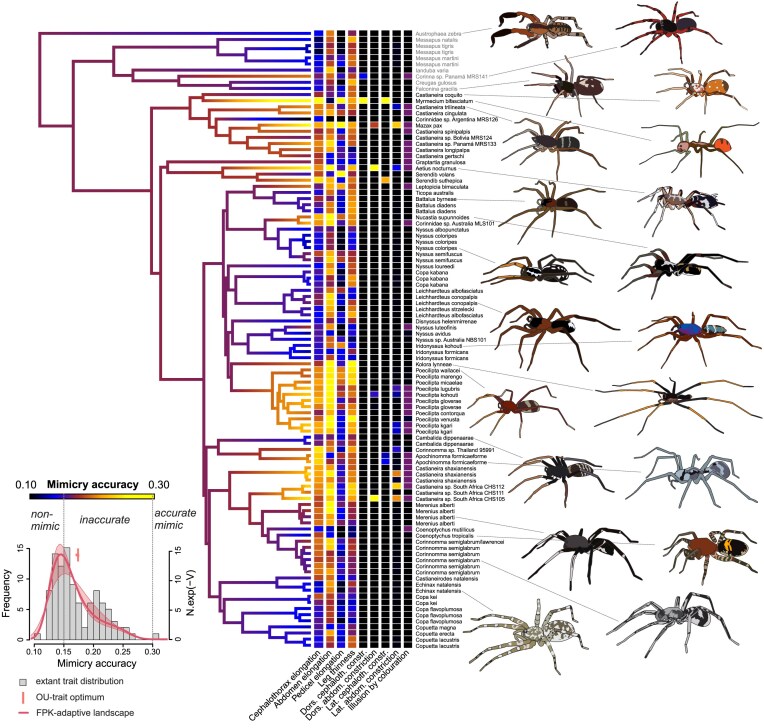
Evolution of ant mimicry in Castianeirinae. Colored branches indicate the evolution of myrmecomorphy strength (mimicry accuracy), with estimation of trait values at internal nodes based on OU-model fit. Colored squares next to the tips indicate the expression of nine traits contributing to the mimicry score. Spider drawings based on live photos of conspecifics or close congeners. The plot in the bottom left shows a histogram of the observed trait variation (in gray), the estimated trait optimum of the fitted OU models across a sample of 100 phylogenies (median indicated by a vertical red bar), and the shape of the adaptive landscape of the fitted FPK model (thick red line for consensus tree, thin lines for the most extreme parameters found across the sample of 100 trees). Outgroup taxa are in gray font, ingroup taxa are in black font.

**Figure 3.**
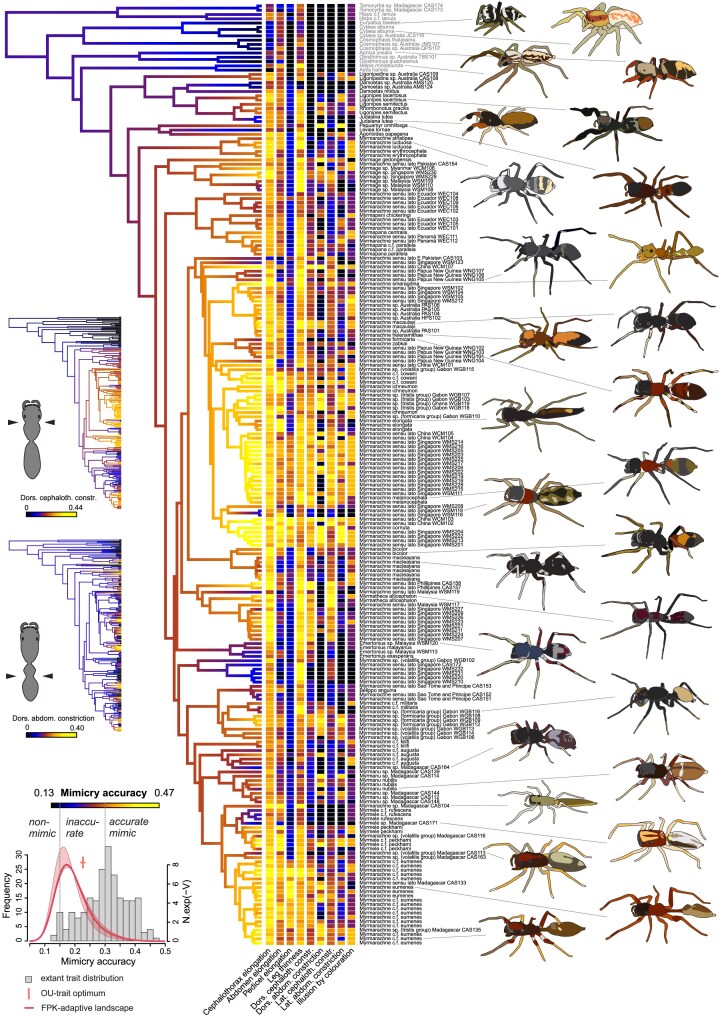
Evolution of ant mimicry in Myrmarachnini. Colored branches indicate the evolution of myrmecomorphy strength (mimicry accuracy), with estimation of trait values at internal nodes based on OU model fit. Colored squares next to the tips indicate the expression of nine traits contributing to the mimicry score. Spider drawings based on live photographs of specimen or conspecifics. Drawings of Malagasy *Myrmarachne sensu lato* based on microscopy images of ethanol-preserved specimens, with live posture and coloration estimated. The two small phylogenies to the left show the inferred trait evolution for cephalothorax and abdominal constrictions. The plot in the bottom left shows a histogram of the observed trait variation (in gray), the estimated trait optimum of the fitted OU models across a sample of 100 phylogenies (median indicated by a vertical red bar), and the shape of the adaptive landscape of the fitted FPK model (thick red line for consensus tree, thin lines for the most extreme parameters found across the sample of 100 trees). Outgroup taxa are in gray font, ingroup taxa are in black font.

Overall, the topology of the BI trees for both Castianeirinae (Fig. 2) and Myrmarachnini (Fig. 3) showed high topological congruence with the tree produced by the ML analyses. Minor topological differences include the placement of *Kolora lynneae* in the Castianeirinae ML tree showing a closer relationship to the clade containing *Nyssus loureedi, Copa kabana*, and the *Leichhardteus* spp., whereas the BI tree placed *K. lynneae* closely related to the clade containing the *Poecilipta* species. Additionally, *Poecilipta lugubris* was closely related to *Poecilipta venusta* in the ML tree but closely related to *P. micaelae* in the BI tree and *P. kohouti* was outside the clade containing *P. micaelae, P. contorqua*, and *P. gloverae* in the ML tree, whereas *P. micaelae* was outside the clade that contained *P. kohouti, P. contorqua*, and *P. gloverae* in the BI tree. All other relationships were the same between the ML and BI trees for Castianeirinae.

Topological incongruence between the ML tree and the BI tree for Myrmarachnini (*n* = 220) includes alternative positions for seven individual samples and three small clades. The seven individual samples resulting in alternative phylogenetic relationships include: *Myrmarachne* sp. indet. Madagascar CAS104; *Myrmarachne sensu lato* Singapore WMS217; *Myrmarachne sensu lato* Singapore WMS214; *Myrmarachne sensu lato* China WCM107; *Myrmarachne sensu lato* Singapore WMS227; *Myrmarachne sensu lato* Singapore WMS211; and *Myrmarachne sensu lato* Singapore WMS221. The three small clades resulting in alternative phylogenetic relationships include: 1) a clade of 5 specimens that includes *Myrmarachne formicaria* CRS101, 3 Papua New Guinean individuals, and 1 Australian individual; 2) a 13-specimen clade that includes the specimens *Myrmarachne smaragdina* Australia TPS307 and other Australian and Singaporean samples; and 3) a clade of 4 specimens including the *Myrmarachne sensu lato* Singapore WMS215 and other Singaporean specimens. All other relationships for Myrmarachnini were the same between the ML and BI trees.

*Note*: We tested the *Myrmecium bifasciatum*–*Castianeira coquito* clade for long branch attraction by removing one of the two taxa from the data set and rerunning the analysis to determine if the placement of the taxon would change, then we repeated the process removing only the other taxon. Neither analysis produced any changes to the topology of the tree, which demonstrates that long branch attraction is not affecting the placement of either taxa within the clade. The dated Bayesian phylogenies for both Castianeirinae and Myrmarachnini are shown in Supplementary Figures S3 and S4, respectively.

Our phylogenomic data sets are the largest thus far for myrmecomorphic spiders and provide a much higher resolution of the macroevolution of Castianeirinae and Myrmarachnini. Our results are mostly consistent with the findings of previous smaller scale studies on these two clades that utilized a limited taxon sample and few marker sequences (Pekár et al. 2017; Wheeler et al. 2017; Maddison and Szűts 2019) or morphological characters (Edwards and Benjamin 2009). However, for some genera, previous phylogenetic hypotheses were not confirmed by our results. Also, there are many undescribed or unidentified specimens in the Myrmarachnini phylogeny. It was beyond the scope of this study to revise the taxonomy of this large clade. Most genera, from which numerous species were utilized, formed monophyletic groups including *Copuetta, Coenoptychus, Poecilipta, Iridonyssus, Leichhardteus, Battalus*, and *Serendib* in the Castianeirinae tree, and *Damoetas, Ligonipes, Emertonius*, and *Myrmage* in the Myrmarachnini tree. In contrast, *Castianeira, Copa*, and *Nyssus* were found to be nonmonophyletic. Although the phylogenetic distance between the *Nyssus* species appears relatively close, the distance between the *Copa* and *Castianeira* species is much larger, appearing in vastly separated clades. Close visual inspection of the sequence alignments was performed to exclude obvious alignment or sequencing errors.

The Myrmarachnini tree included many undescribed species with unclear taxonomy making a check of genus monophyly less clear, but we found that such specimens originating from similar geographic regions usually formed distinct clades. The large representation of Malagasy samples formed a single clade except for three individuals that grouped together in a more distant clade. The South African and Gabonese species formed two distant clades with two South African exceptions that diverged early in the Malagasy clade and one Gabonese sample diverging early in a Singaporean clade. The Malaysian and Singaporean samples, coming from regions in very close proximity to each other, formed numerous clades together. The South American, Australian, and Papua New Guinean species also formed several distinct clades.

For Castianeirinae, Wheeler et al. (2017) found a closer phylogenetic relationship between *Serendib* and *Myrmecium*, albeit with low support. Our study places *Serendib* more closely aligned with *Aetius* with maximal support (UBS = 100%). Additionally, Wheeler et al. (2017) found that *Corinnomma* was closely related to *Nyssus*, which is not supported by our study. For Myrmarachnini, Edwards and Benjamin (2009) conducted a phylogenetic analysis using morphological characters and produced phylogenetic relationships mostly congruent with this study. The phylogenetic relationships between numerous Australian members of the genus *Myrmarachne* have been established previously using Sanger sequencing of two gene fragments (Pekár et al. 2017). The close phylogenetic relationships shared by *Myrmarachne macleayana* and *Myrmarachne bicolor*, those shared by *M. smaragdina, M. helensmithae*, and *M. macaulayi*, as well as those between *M. striatipes, M. lu ctuosa*, and *M. erythrocephala* reported by Pekár et al. (2017) are also supported here. The results of our study also largely support those produced in a more recent study of Myrmarachnini phylogeny that used three gene regions (28S rRNA, 16S rRNA region ND1, and cytochrome *c* oxidase subunit I; Maddison and Szűts 2019).

### Comparative Analyses

The likelihood fit of the Castianeirinae and Myrmarachnini UCE phylogenetic data and the mimic accuracy indices, as well as the nine selected myrmecomorphic traits, were analyzed using BM (“random walk”), OU (constrained evolution), and EB (early adaptive radiation) evolutionary models. The FPK model that was used as an additional, more complex model allowing for a multimodal adaptive landscape, was not used for ancestral character estimation as in tests the ace function implemented in the *BBMV* package did not return realistic node states. In tests, the ML estimated adaptive landscape of fitted FPK models showed a single optimum for most traits, which approximates an OU model. For most traits of the alternative models BM, OU, and EB distributions were best fitted with OU models (Table 2). Accordingly, the input (consensus) tree was alpha transformed before the estimation of node states, using the alpha of the fitted model in *geiger*. In Castianeirinae, the phylogenetic distribution of two traits (“Lateral cephalothorax constriction” and “Dorsal cephalothorax constriction”) was best explained with an EB model. In these cases, the tree was transformed by the EB model parameter *a*. Only one trait, “Dorsal abdominal constriction,” was best fitted with a BM model, and hence, in this case, the input tree was left unchanged for the estimation of node states.

**Table 2.** AICcw values based on the consensus tree for the Castianeirinae and Myrmarachnini indicating the relative likelihood fit of the mimic accuracy data and the nine selected myrmecomorphic traits using BM, OU, and EB models of evolution.

	Model type		
Characteristic	BM	OU	EB
**Castianeirinae**			
Thin legs	0.000	**1.000**	0.000
Elongation of cephalothorax	0.000	**1.000**	0.000
Elongation of abdomen	0.000	**1.000**	0.000
Elongation of pedicel	0.000	**1.000**	0.000
Lateral constriction of cephalothorax	0.046	0.016	**0.938**
Dorsal constriction of cephalothorax	0.000	0.000	**1.000**
Lateral constriction of abdomen	0.000	**1.000**	0.000
Dorsal constriction of abdomen	0.182	**0.755**	0.063
**Myrmarachnini**			
Thin legs	0.000	**1.000**	0.000
Elongation of cephalothorax	0.004	**0.994**	0.002
Elongation of abdomen	0.000	**1.000**	0.000
Elongation of pedicel	0.000	**1.000**	0.000
Lateral constriction of cephalothorax	0.001	**0.999**	0.000
Dorsal constriction of cephalothorax	0.001	**0.999**	0.000
Lateral constriction of abdomen	0.000	**1.000**	0.000
Dorsal constriction of abdomen	0.000	**1.000**	0.000

Note: The favored model (highest value) is in bold.

### Overall mimicry accuracy

**Figure 4.**
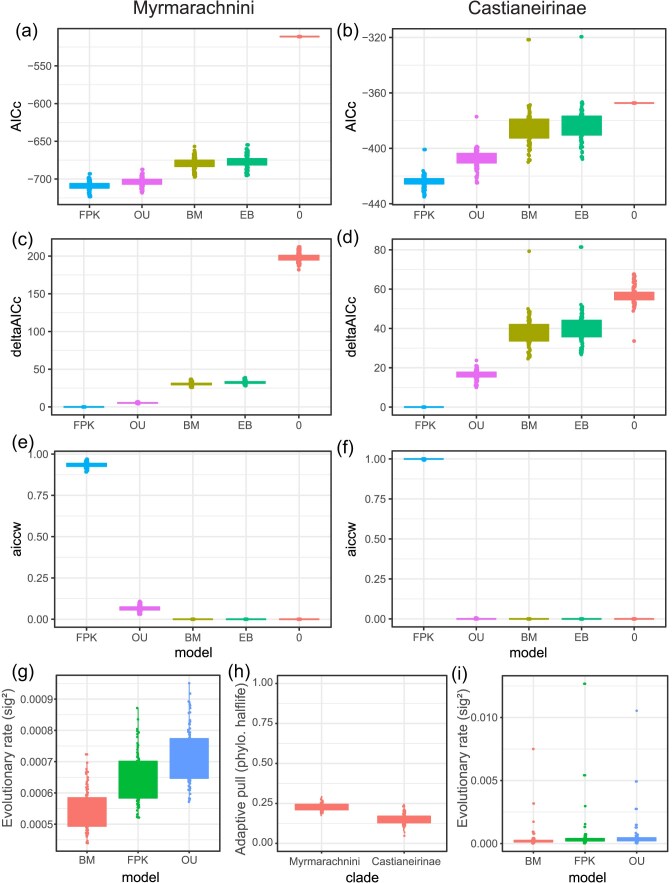
Phylogenetic sensitivity of evolutionary model fit and model parameters of mimicry accuracy evolution in Myrmarachnini (a, c, e, g) and Castianeirinae (b, d, f, i). a) and b) Comparison of the AICc for five alternative evolutionary models: BM; OU; EB; FPK; and 0, white noise. c) and d) Delta AICc values. e) and f) AICcWs. g) and i) Evolutionary rate of the fitted BM, OU, and FPK models. h) Phylogenetic half-life calculated from the alpha of fitted OU models relative to the total height of the phylogenetic tree.

The inferred phylogenetic structure of trait expressions (Figs. 2 and 3) supports the theory that accurate mimicry evolves through the gradual and accumulating changes of myrmecomorphic traits. Ancestral character estimation indicates that within each of the myrmecomorphic groups, the moderately accurate extant species have diverged from inaccurate ancestors, and in Myrmarachnini, accurate extant species have diverged from moderately accurate ancestors. In our Castianeirinae taxon sampling, only a single species was highly accurate, whereas in Myrmarachnini, high overall mimic accuracy was scattered throughout the tree with the highest frequency in two clades. Despite support for a gradual evolutionary process, parameter estimates for both OU and FPK models indicated that higher levels of mimetic accuracy are at an unstable state, which was acquired and lost multiple times independently (Figs. 2 and 3 insets and Fig. 4). Therefore, the perfecting hypothesis does not explain inaccurate mimicry due to the unstable nature of accurate mimicry, thus we cannot infer which direction selection is driving mimic species—that is, either maintaining mimic inaccuracy, evolving toward higher levels of accuracy or perhaps even the complete loss of mimetic traits.

### Myrmecomorphic trait distribution

Thin legs were a typical trait for all Myrmarachnini (Supplementary Fig. S5) with only a few lineages scattered across *Myrmarachne sensu lato* exhibiting reversed evolution toward thicker legs. Castianeirinae showed evolution around moderate leg thickness, with separate lineages evolving toward thinner legs and others toward thicker legs. In Castianeirinae, an elongated cephalothorax evolved repeatedly in distinct lineages, often with sister clades showing opposite trends. An elongated cephalothorax emerged at the base of Myrmarachnini and evolved gradually longer toward the core clade. Abdominal elongation evolution was dynamic in both clades, often isolated in single lineages or species. In Castianeirinae, pedicel elongation was scattered across the clade, with multiple independent events in separate lineages or species. Pedicel elongation evolved patchily in Myrmarachnini, with three origins in one clade composed of *Myrmarachne sensu lato* from Southeast Asia and Africa, and another case in a distantly related clade from Madagascar.

### Constrictions of the cephalothorax

Lateral cephalothorax constriction evolved early in the evolutionary history of the *Myrmarachne sensu lato* group, has been enhanced (greater constriction) in several clades, particularly in individuals collected from Singapore, but has been lost in groups from Malaysia and Madagascar. However, in the Malagasy clade constrictions have subsequently re-evolved. In Castianeirinae, the lateral constrictions of the cephalothorax have only evolved in three lineages, most notably *Myrmecium* and *Serendib*. The lateral constrictions of the cephalothorax were closely tied with the evolution of the dorsal constriction in Myrmarachnini and two of the Castianeirinae, *Myrmecium* and *Serendib*.

### Constrictions of the Abdomen

In Myrmarachnini, abdominal constrictions were more dynamic and scattered across separated lineages compared with cephalothorax constrictions with dorsal and lateral constrictions of the abdomen correlated. In Castianeirinae, abdominal constrictions evolved only in a few nonrelated lineages, with dorsal and lateral abdominal constrictions evolving independently, except for a *Castianeira* sp. from South Africa. For further discussion of all the individual myrmecomorphic traits, see online Appendix 1 (II. Myrmecomorphic Trait Evolution).

### Phylogenetic Signal Analysis

**Figure 5.**
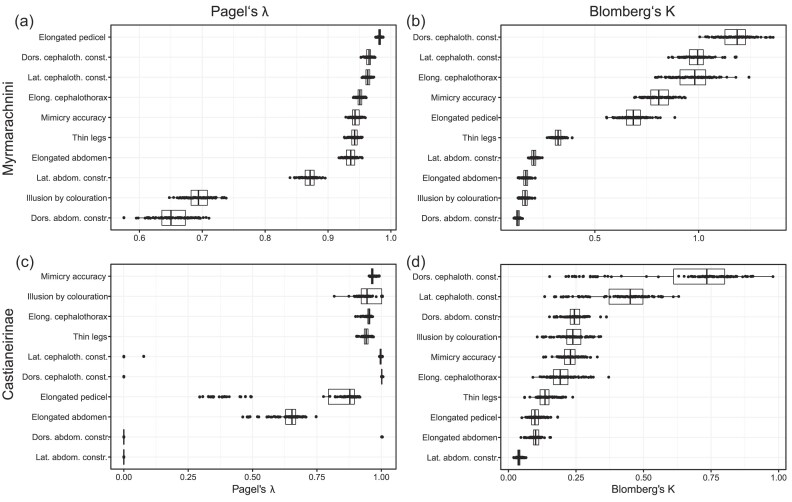
Phylogenetic signal across sample of 100 trees for each Myrmarachnini (a and b) and Castianeirinae (c and d). a) and c) Pagel’s lambda; b) and d) Blomberg’s *K*. Note that estimates for some traits in Castianeirinae must be interpreted with caution due to insular trait expression: “Lateral cephalothorax constriction” (Lat cephaloth. const.), “Dorsal cephalothorax constriction” (Dors. cephaloth. const.), “Lateral abdomen constriction” (Lat. abdom. constr.), “Dorsal abdomen constriction” (Dors. abdom. constr.), and “Illusion by colouration”. The high abundance of nonexpression returns a very high or very low phylogenetic signal for those traits.

To understand the dynamics of the traits underlying mimicry accuracy, the phylogenetic signal was analyzed for each trait (Fig. 5). In Myrmarachnini, the elongated pedicel, thin legs, and the dorsal and lateral cephalothorax constrictions had strong phylogenetic signal (i.e., phylogenetically stable; Fig. 5a). In contrast, lateral and dorsal constrictions of the abdomen had low phylogenetic signal (i.e., were phylogenetically labile). Elongation of the abdomen was less stable than elongation of the cephalothorax.

For Castianeirinae, interpretation of the phylogenetic signal was limited, as some trait distributions strongly deviated from normal due to nonexpression in the majority of lineages (Fig. 5c), with the exception of thin legs that were evolutionarily stable.

### Phylogenetic Sensitivity Analysis

We further investigated the phylogenetic sensitivity of the inferred evolutionary model fit and parameter estimates and found that results were highly consistent across the tree sample. The FPK model had the best fit, followed by the OU model (Fig. 4a–f). Inspection of the inferred model parameters and the polynomial functions describing the adaptive landscape of the FPK model showed high consistency across alternative trees in both clades, with a single optimum at a mimicry accuracy score between 0.13 and 0.15 in Castianeirinae (Fig. 2 inset) and between 0.15 and 0.18 in Myrmarachnini (Fig. 3 inset). In both clades, the curve was slightly skewed toward lower accuracy, but otherwise comparable to the adaptive landscape of an OU model (Boucher et al. 2018). Compared with the FPK model, the fitted OU models exhibited slightly higher trait optima (Figs. 2 and 3 insets) and slightly higher evolutionary rates (Fig. 4g and i).

The evolutionary rate (*σ*²) and *α* of the fitted OU models were comparable in both clades. The phylogenetic half-life calculated from *α* indicates how fast the trait evolves toward the trait optimum *θ*. With a value of 5th–4th of the total tree height, mimicry accuracy had a low phylogenetic half-life in both Myrmarachnini and Castianeirinae (Cooper et al. 2016), with slightly lower values in the latter. Because the inferred trait optimum *θ* is at a low accuracy level and the phylogenetic half-life is low, mimicry accuracy tends to converge toward an inaccurate state from both nonmimicry and highly accurate mimicry at high rates and speed. This indicates that mimicry accuracy is generally an evolutionary labile trait and that strong myrmecomorphy is a macroevolutionary highly unstable state.

## Discussion

### The Evolution of Batesian Mimicry Converges Toward Inaccurate Expression

In this comprehensive macroevolutionary study of myrmecomorphy in spiders, we tested the hypothesis that inaccurate mimicry represents unstable transitional stages (on the macroevolutionary scale) and that adaptative peaks are found at highly accurate mimicry. We did not find support for this hypothesis, but instead the results indicate that inaccurate mimicry represents an adaptive optimum, whereas highly accurate states are evolutionary labile. We found evidence that accurate states, where present, evolved from inaccurate states in a gradual or step-by-step manner, by the accumulation and enhanced expression of multiple traits contributing to myrmecomorphy. This has previously been suggested for the evolution of myrmecomorphy in spiders at the family level (Pekár 2014) and for Batesian mimicry in coral snakes (Kikuchi and Pfennig 2010).

However, we also found that the evolution of myrmecomorphy is highly dynamic and that trends toward higher mimicry accuracy are frequently reversed, similar to wasp-mimic hoverflies (Leavey et al. 2021). Our results suggest that inaccurate states are not transitional states per se but may more frequently represent an evolutionary optimum; therefore, we find no support for the perfecting hypothesis as an explanation for inaccurate mimicry. Both OU and FPK models indicated the same global pattern: that integrated selection globally favors inaccurate mimicry and that accurate mimicry is phylogenetically unstable. This aligns with the common observation of inaccurate mimicry across many taxa and the development of novel theoretical frameworks (Dalziell and Welbergen 2016). Although we found myrmecomorphy to be expressed differently in the two focal clades, the rates and modes of mimicry evolution were comparable between them (Myrmarachnini and Castianeirinae). One caveat to our overall finding is that the single accurate Castianeirinae species included in our analyses (*M. bifasciatum*) was sister group to a low accuracy species (*C. coquito*)—but as both were divided by long branches and nested within a clade of predominantly inaccurate mimics. It is most plausible that *Myrmecium* evolved the high myrmecomorphy via a gradual process, but our sampling may not have included the intermediate sister species between these specimens. Alternatively, a punctuated model is also possible in this case.

Our overall results were robust against uncertainties in phylogenetic topologies and divergence time estimates, indicating that the evolution of myrmecomorphy follows the same rules in different clades of spiders that acquired ant mimicry independently. Inaccurate Batesian mimicry has been recorded frequently (e.g., spiders, hoverflies, butterflies, and snakes) suggesting that the adaptive peak may be inaccurate mimicry (Ruxton et al. 2019). Our study is the first attempt at a large-scale phylogenetic comparative approach supporting that this might be a global pattern.

### Ecological Effects on Myrmecomorphic Trait Expression

#### Proximity to models

There is evidence of relaxed selection on mimetic traits when models are rare or absent, such as in coral snake mimicry (Davis Rabosky et al. 2016; Hodson and Lehtinen 2017). Equally, proximity to ants may play a role in mimic accuracy in myrmecomorphic spiders (Edmunds 1978; Edmunds 2006; Cushing 2012), but not to the point of predators being ant-naïve because ants are found throughout almost all terrestrial habitats.

#### Clade-specific environmental context

Some of the differences observed between corinnid sac spiders (e.g., Castianeirinae) and jumping spiders (e.g., Myrmarachnini) may be explained by their predominant ecology. Most jumping spiders are found in the vegetation and other aboveground habitats (Cumming and Wesołowska 2004), whereas most corinnid sac spiders are mostly ground-, bark-, and litter-dwelling. Thus, the occupation of aboveground habitats may lead to greater exposure to predators and hence greater selective pressures to evolve defensive strategies (Perger et al. 2022), whilst crypsis might be more successful in ground- and bark-dwelling corinnid spiders. A case in point is the accurate corinnid ant mimic *Myrmecium* that is frequently found on the vegetation (Candiani and Bonaldo 2017). Evolution toward either aposematism/mimicry or crypsis depending on habitat choice is also known from other animals, such as butterflies and fish (Poole 1970; Cortesi et al. 2016; Willmott et al. 2017).

#### Trade-offs

The constrictions to the abdomen were among the phylogenetically most labile traits, possibly due to an apparent trade-off with fecundity, in terms of egg production in females (Cushing 1997; Sarikaya et al. 2019). Cephalothorax constrictions were evolutionarily more stable traits, but the magnitude of the constriction could result in a trade-off with negative effects on hemolymph hydraulics which may affect jumping capabilities (Shamble et al. 2017; Hashimito et al. 2020) and brain size (i.e., cognition; Kelly et al. 2024). Although corinnids are not renowned for their cognition or jumping, cephalothorax constrictions and elongations were rare in this clade, but when they occur (e.g., *Myrmecium*) they are often extreme (Candiani and Bonaldo 2017) compared with Myrmarachnini.

#### Motion-limited discrimination

The overall greater resemblance to ants in the salticids compared with the corinnids could be explained by differences in running speed, whereby corinnids are often noted for their fast-running speed (Perger and Pett 2022). Predators may not be able to distinguish less-accurate mimics from models if they move at speed (“motion-limited discrimination”). Although one study found weak support indicating myrmecomorphic accuracy is negatively related to speed in analyses that considered corinnids, salticids, and insect ant mimics (Pekár et al. 2022), another study reported no support (McLean and Herberstein 2021). It is notable that both studies considered a range of spider and insect myrmecomorphs collectively, therefore we are unable to compare running speeds between salticids and corinnids specifically, which has intriguing potential.

The dynamics of mimicry evolution we found in our study indicate that ecology-specific selection pressures and trade-offs may act differently on the compound traits that contribute to the highly integrative phenotype of a myrmecomorphic body. The result is a dynamic adaptive landscape where locally either inaccurate or accurate mimicry provides the best fitness benefits. Setting aside that ants themselves can be morphologically variable (see Andersen 2016), potential local factors that might contribute to these dynamics include the loss of the model (e.g., Prudic et al. 2002) or changes in predation pressure due to the availability of alternative prey (Kikuchi et al. 2022).

## Conclusion

Here, we have shown that contrary to the prediction that strong selection from predators should result in increased mimic accuracy over evolutionary time, accurate mimicry is a highly unstable state on the macroevolutionary scale and frequently converges toward inaccurate mimicry. Our multitaxon approach revealed a complex assembly of selection targets and trade-offs, that may explain why accurate mimicry is rarely maintained. This result thus reverses the onus of explanation from inaccurate mimicry to explaining the exceptional evolution and maintenance of accurate mimicry.

## Diversity and Inclusion Statement

We are strong supporters of equity, diversity, and inclusion in sciences (Rößler et al. 2020). Although our list of authors is heavily male-biased, we stem from diverse cultural backgrounds and represent different stages of research careers, from Ph.D. student to Professor and at least one of the authors identifies as a member of the LGBTIQA+ community.

## Supplementary Material

syaf037_Supplemental_Files

## Data Availability

Online Appendices 1, 2, and 3 and Supplementary figures and tables are available at Zenodo (https://zenodo.org/records/14192554).

## Supplementary Material

Data and code available from Dryad Digital Repository: https://doi.org/10.5061/dryad.18931zd45.
