# Supplementary material for: Batesian Mimicry Converges toward Inaccuracy in Myrmecomorphic Spiders
Source: Syst Biol. 2025 May 19;74(6):967–84. doi: 10.1093/sysbio/syaf037 (PMC12712336; doi:10.1093/sysbio/syaf037)
Supplement: syaf037_Supplemental_Files [file syaf037_supplemental_files.zip › Appendix 1.pdf]

Appendix 1

I. SPECIES IDENTIFICATION

Remarks on species identification. Myrmarachnini are a very diverse group with, by this date, highly incomplete taxonomy. This makes the identification of some specimens difficult, especially from Africa and Madagascar. For the reasoning behind the ID used in this study for problematic terminals see our remarks below.

MADAGASCAR

- *Hispo* sp. Madagascar CAS175 and CAS176 propose *Hispo* cf. *tenuis* Wanless, 1981. The markings are similar to the author's illustration but slightly larger in our specimen, but note that the distribution of this species is unclear from the original description. The species is indicated in the key, abstract, and distribution as being from Madagascar, but the type material examined is only from Sri Lanka. Also, the females of most *Hispo* species are undescribed, including *tenuis*.

- *Tomocyrba* sp. Madagascar CAS173 - *Tomocyrba* n. sp.? Epigyne does not agree with any of the known species, so it is probably an undescribed species.

- *Myrmarachne sensu lato* Madagascar CAS104 - cannot be identified further as it is a juvenile.

- *Myrmarachne sensu lato* Madagascar CAS106 - male, probably related to *Myrmarachne* cf. *cowanii* (Peckham and Peckham, 1892), with a longer pedicel than most, but not that species (possibly new).

- *Myrmarachne sensu lato* Madagascar CAS110 - *Myrmarache eumenes* (Simon, 1900) female. Confident ID as the epigyne is a good match due to the long petiole. Also, this species was recorded from several localities not too far from this specimen's locality. Although Wanless never proposed a group for *M. eumenes* (considering it species sola), we refer to this species and close relatives as the eumenes group here, for practicality.

- *Myrmarachne sensu lato* Madagascar CAS112 - *Myrmanu* n. sp.? This specimen has a very distinctive RTA unlike anything in Wanless' revision, but it groups together with the nubilis group, whose males are unknown, so we consider this a member of that group.

- *Myrmarachne sensu lato* Madagascar CAS113 - Probably a new species, a member of the volatilis group. Quite distinct epigyne structure in this group (round atria, subtriangular pouch present), but the copulatory ducts are longer than any of the described species.

- *Myrmarachne sensu lato* Madagascar CAS114 - *Myrmanu* n. sp.? Clearly related to CAS112, similar RTA, but abdomen shape and markings are different. It also groups together with the nubilis group in the tree.

- *Myrmarachne sensu lato* Madagascar CAS115 - *Myrmarachne* cf. *cowanii* (Peckham & Peckham, 1892), couldn't ID definitively because the epigyne is not clear, but morphologically it is very close to this species.

- *Myrmarachne sensu lato* Madagascar CAS116 - This species probably belongs to the volatilis group, also having round atria and a subtriangular pocket, but the epigyne is not clear enough. In the tree it is quite far from CAS113, although in the same large clade, whose taxonomy is unresolved. It could represent a very large and well-represented species group.

- *Myrmarachne sensu lato* Madagascar CAS117 and CAS118 - Very reminiscent of *M. eumenes*, with a long petiole. Probably new.

- *Myrmarachne sensu lato* Madagascar CAS119 - *Myrmele rufescens* (Simon, 1900). Redescribed and renamed *Myrmarachne eugenei* by Wanless, it was transferred to *Myrmele* by Prószyński (2016). Of the four species in

this genus, this one has the most indistinct carapace constriction. This specimen is also from the type locality (Diego Suarez = Antsiranana), so this ID is fairly certain.

- *Myrmarachne sensu lato* Madagascar CAS120 - male, probably related to *M. Eumenes*, at least appears like the same species morphologically, but they are in very different clades in the tree.

- *Myrmarachne sensu lato* Madagascar CAS121 - female of 120, so at least they group together in the tree. The epigyne is remarkably similar to Wanless' drawings of *M. eumenes*, but the markings are clearly different, and this species is in a clade sister to the one containing CAS110 *M. eumenes*.

- *Myrmarachne sensu lato* Madagascar CAS122 (cf. *cowanii* (Peckham & Peckham, 1982)) - the male of CAS 115, from the same locality and also closely matching the description, but palps could not be checked thoroughly.

- *Myrmarachne sensu lato* Madagascar CAS124 - male, possibly related to *Myrmarachne augusta* (G. W. Peckham & E. G. Peckham, 1892), not assigned to a particular group.

- *Myrmarachne sensu lato* Madagascar CAS125 - This looks like *M. eumenes* (Simon, 1900), and is very similar to CAS110. They also fall within the same clade with a small genetic distance, and from the same locality, so likely conspecifics.

- *Myrmarachne sensu lato* Madagascar CAS126 - Also *M. eumenes* (Simon, 1900), has the two small denticles on the dorsal side of the chelicerae too.

- *Myrmarachne sensu lato* Madagascar CAS128 - *Myrmele* sp., close to *rufescens*, if not the same species. This specimen is sister to female CAS119, with a short genetic distance, except the markings are different, but they are from the same locality, so this is probably the conspecific male.

- *Myrmarachne sensu lato* Madagascar CAS129 and CAS130 - related to *M. eumenes* (Simon, 1900), with a similar general epigyne shape and long petiole. Female and male, respectively, falling together with CAS117 and 118.

- *Myrmarachne sensu lato* Madagascar CAS133 - *M. eumenes* (Simon, 1900) male based on somatic and genitalic characters and position in the tree.

- *Myrmarachne sensu lato* Madagascar CAS134 - female, probably *Myrmanu nubilis* (Wanless, 1978). Somatic morphology similar to CAS136 below, which was identified as *M. nubilis*, but this epigyne is not clear. Genetic distance is small, indicating that they are conspecific.

- *Myrmarachne sensu lato* Madagascar CAS135 - male, belonging to the tristis group, which has a hooked RTA plus an additional lateral flange. The "problem" is that this group hasn't been recorded from Madagascar, and all Wanless' species are from continental Africa. It looks very similar to several species from South Africa.

- *Myrmarachne sensu lato* Madagascar CAS136 - *Myrmanu nubilis* Wanless, 1978, female. The vulva closely matches his drawing, particularly the subtriangular pouch extending to the epigastric fold and the ovoid atria, and the specimen is also about 350 km N of the type locality in a similar coastal forest.

- *Myrmarachne sensu lato* Madagascar CAS137 - related to *M. eumenes* (Simon, 1900); similar shape and long petiole, and falls into an adjacent clade.

- *Myrmarachne sensu lato* Madagascar CAS138 - related to *M. eumenes* (Simon, 1900), female of CAS137, from the same locality.

- *Myrmarachne sensu lato* Madagascar CAS139 - closely related to *Myrmanu nubilis*. Although the epigyne is unclear, the subtriangular pocket is present and seems to run to the epigastric fold, and it falls in a clade with *nubilis*.

- Myrmarachne sensu lato Madagascar CAS140 - *Myrmele* sp., close to *M. rufescens*. The epigyne is quite close to *M. eugenei* [= *rufescens*] with similar morphology, so likely it is the same species.

- *Myrmarachne sensu lato* Madagascar CAS141 - *Myrmarachne* cf. *augusta* (G. W. Peckham & E. G. Peckham, 1892). Similar chelicerae and somatic morphology to CAS124, but abdomen setae are golden while white in CAS124, so it is probably a different related species.

- *Myrmarachne sensu lato* Madagascar CAS142 and CAS143 - *Myrmele peckhami* Roewer, 1951. Morphology consistent, palp not clearly visible, but epigyne a perfect match. Both are from the same locality, so pretty sure they both belong to this species.

- *Myrmarachne sensu lato* Madagascar CAS144 - *Myrmanu* sp. n.? Similar to CAS112 and 114, this species has a slender RTA and lateral flange, and falls in the same clade.

- *Myrmarachne sensu lato* Madagascar CAS146 - *Myrmarachne* cf. *eumenes* (Simon, 1900), female of this group and matched to CAS147, from the same locality.

- *Myrmarachne sensu lato* Madagascar CAS147 - *Myrmarachne* cf. *eumenes* (Simon, 1900), clearly a male of this group.

- *Myrmarachne sensu lato* Madagascar CAS148 - *Myrmanu* sp. n.? This is the matching female of the male CAS112. Same locality and group together in the tree.

- *Myrmarachne sensu lato* Madagascar CAS149 - *Myrmele* cf. *peckhami* Roewer, 1951. It has a very similar body form to *M. peckhami*, but the epigyne is not clear so we cannot make a definitive ID.

- *Myrmarachne sensu lato* Madagascar CAS150 - *Myrmanu nubilis* (Wanless, 1978), epigyne a good match and groups with other *nubilis* specimens.

- *Myrmarachne sensu lato* Madagascar CAS160 - related to *eumenes* (Simon, 1900), female, possibly the same species as CAS137 and 138, but from a different locality.

- *Myrmarachne sensu lato* Madagascar CAS161 - *Myrmele* cf. *peckhami* Roewer, 1951, male, cannot see palp properly but possibly *M. peckhami*, coloration very similar.

- *Myrmarachne sensu lato* Madagascar CAS162 - *Myrmarachne* cf. *eumenes* (Simon, 1900). Male, similar to CAS147 morphologically, but orange in color rather than brown. In the same larger clade, but the three specimens (CAS146, CAS147 and CAS162) are separated by an anomaly from the tristis group.

- *Myrmarachne sensu lato* Madagascar CAS163 - Probably a new species, a member of the volatilis group, with a similar carapace to CAS113 but clearly different abdominal markings. Quite distinct epigyne structure in this group (round atria, subtriangular pouch present), but the copulatory ducts are longer than any of the described species, even CAS113.

- *Myrmarachne sensu lato* Madagascar CAS164 - *Myrmarachne* sp. indet., female. An unusual species, with long setae over the entire body, but the epigyne is not clear. It falls in a clade with *M.* cf. *augusta*, who is the sole representative in its "group", whose females are unknown. It could be related, but the genetic distance is fairly long, so all of these species in this clade (CAS124, CAS141, CAS164, CAS170 and possibly CAS145 from Comoros, too) might be a single species group.

- *Myrmarachne sensu lato* Madagascar CAS165 - *Myrmarachne* cf. *eumenes* (Simon, 1900). Male, similar to CAS162, but dark brown, chelicerae shorter and dorsal teeth less distinct. Possibly just variation.

- *Myrmarachne sensu lato* Madagascar CAS166 - *Myrmele* cf. *peckhami* Roewer, 1951, similar to the others in this clade, but dorsal teeth on chelicerae even larger.

- *Myrmarachne sensu lato* Madagascar CAS170 - *Myrmarachne* cf. *augusta* (G. W. Peckham & E. G. Peckham, 1892). Clearly related, with similar cheliceral morphology, but the coloration is quite distinct from others in this clade.

- *Myrmarachne sensu lato* Madagascar CAS171 - *Myrmele* sp.n. Body shape and epigyne very similar to *M. rufescens*, but the markings are totally different.

COMOROS

- *Myrmarachne sensu lato* Comoros CAS145 - *Myrmarachne* cf. *augusta* (Peckham & Peckham, 1892). This female has similar somatic and epigynal morphology to *M. augusta*, but very different coloration, so probably a new species. Incidentally, Wanless indicated that the genus was absent from the Comoros and has not been subsequently recorded, so this is a major find.

SÃO TOMÉ & PRÍNCIPE

- *Myrmarachne sensu lato* São Tomé and Príncipe CAS151, CAS152, CAS153 - *Belippo*/*Myrmarachne* sp. indet. This is a really challenging specimen, because it groups together with CAS168. Their morphology is very different, with these three specimens resembling females of *M.* cf. *augusta*, but that makes little sense biogeographically. If they are *Myrmarachne* and not *Belippo* then probably belong in the same species group as *augusta*.

- *Myrmarachne sensu lato* São Tomé and Príncipe CAS168 - This specimen matches the dorsal illustrations and markings of *Belippo anguina* Wanless, 1978, but the epigyne is too unclear to make a definitive ID. The locality is 12km from the type locality, hence they are probably the same species.

GABON

- *Myrmarachne sensu lato* Gabon WGB101 - *Myrmarachne* cf. *militaris* Szombathy, 1913. It is probably this species, but the palp could not be checked. The thoracic region of the male is broader than in *M. lawrencei*, which also occurs in Gabon.

- *Myrmarachne sensu lato* Gabon WGB102 - *Myrmarachne* sp. (volatilis group). This species may be related to *M. globosa*, whose epigyne it resembles most closely, but we really cannot be sure. The latter species is also distributed in west-Central Africa, so it is plausible. It seems to have a dorsal scutum covering the entire abdomen, which is unusual. Quite isolated in the tree, so not absolutely sure of its affinities.

- *Myrmarachne sensu lato* Gabon WGB103 - *Myrmarachne* sp. (tristis group). There are many continental African species in this group and require palpal examination to confirm their identification. Several species occur in Gabon and more potentially here, so impossible to ID without detailed examination.

- *Myrmarachne sensu lato* Gabon WGB104 and WGB105 - *Myrmarachne elongata* Szombathy, 1915, male and female. Widespread in West and Central Africa from Angola northwards, this male might belong to this species but requires palpal examination. However, the genetic distance to WGB105 is small and they are from the same locality, and its epigyne is a good match to Wanless' drawings of *M. elongata*, so we are fairly confident in the ID.

- *Myrmarachne sensu lato* Gabon WGB105 - *Myrmarachne* sp. (volatilis group). This species may be related to *M. globosa*, whose epigyne it resembles most closely, but we really cannot be sure. The latter species is also distributed in west-Central Africa, so it is plausible. It seems to have a dorsal scutum covering the entire abdomen, which is unusual. Quite isolated in the tree, so not absolutely sure of its affinities.

- *Belippo* sp. Gabon WGB106 - *Myrmarachne* sp. (volatilis group). The epigynal morphology is inconsistent with *Belippo* but is similar to members of the volatilis group of *Myrmarachne*.

- *Myrmarachne sensu lato* Gabon WGB107 - *Myrmarachne* sp. (tristis group) male, but palp could not be checked.

- *Myrmarachne sensu lato* Gabon WGB108 - *Myrmarachne* sp. (formicaria group). A challenging species to ID without genitalia, but the carapace shape is most similar to members of this group.

- *Myrmarachne sensu lato* Gabon WGB109 - *Myrmarachne* sp. (formicaria group). The carapace shape is most similar to members of this group, and it falls in the same clade as the previous species.

- *Myrmarachne sensu lato* Gabon WGB110 - *Myrmarachne* sp. (tristis group). The epigyne lacks a subtriangular pocket and the atria are subtriangular, so it fits in this group well, but the epigyne is unclear so we cannot make a definitive ID.

- *Myrmarachne sensu lato* Gabon WGB111 - *Myrmarachne elongata* Szombathy, 1915, male morphologically and genetically similar to WGB104 and WGB105.

- *Myrmarachne sensu lato* Gabon WGB112 - *Myrmarachne* sp. (formicaria group). The carapace shape and coloration are similar to WGB108 and WGB109 above.

- *Myrmarachne sensu lato* Gabon WGB116 - *Myrmarachne* sp. (formicaria group). Male but indistinguishable.

- *Myrmarachne sensu lato* Gabon WGB117 - *Myrmarachne* cf. *militaris* Szombathy, 1913, female. Epigyne has the correct general form, but is at an oblique angle, so the ID cannot be confirmed.

- *Myrmarachne sensu lato* South Africa WGB120, WGB121 and WGB122 - *Myrmarachne ichneumon* (Simon, 1886), a very distinct species well known by one of the authors (CH) from South Africa.

II. MYRMECOMORPHIC TRAIT EVOLUTION

The evolution and extant distribution of the individual traits contributing to myrmecomorphy (see Fig. S5).

Summary **-** Castianeirinae showed evolution around moderate leg thickness, with separate lineages evolving towards thinner legs and others towards thicker legs. Thin legs were a typical trait for all Myrmarachnini with only a few lineages scattered across *Myrmarachne sensu lato* exhibiting reversed evolution towards thicker legs. In Castianeirinae, an elongated cephalothorax evolved repeatedly in distinct lineages, often with sister clades showing opposite trends. An elongated cephalothorax emerged at the base of Myrmarachnini and evolved gradually longer towards the core clade. Abdominal elongation evolution was dynamic in both clades, often isolated in single lineages or species. In Castianeirinae, pedicel elongation was scattered across the clade, with multiple independent events in separate lineages or species. Pedicel elongation evolved patchily in Myrmarachnini, with three origins in one clade composed of *Myrmarachne sensu lato* from Southeast Asia and Africa, and another case in a distantly related clade from Madagascar.

1) Thin legs.

Castianeirinae - Moderately thin legs appear in the outgroup species and are estimated for deep ancestral nodes indicating that thin legs had evolved early in the evolutionary history of this group. The thin leg trait has seen further refinement in the *Poecilipta* clade and a few of the *Castianeira* species and has been reduced or lost in the genera *Castianeirodes*, *Echinax*, *Graptartia*, *Iridonyssus*, *Merenius*, *Coenoptychus*, many *Nyssus* and *Copa* species, some *Copuetta*, *Castianeira* and *Leichhardteus* species, and *Corinnomma semiglabrum*.

Myrmarachnini - The trait of thin legs was estimated in this study to have evolved into a moderate to higher level of ‘leg thinness’ in the earliest ancestors of this group. In most cases, the trait has evolved to very thin legs

with several occurrences of the leg evolving to moderate leg thinness and several occurrences of the reversion where the leg has increased in thickness relative to the estimated ancestral condition (e.g., *Damoetas* sp.).

2) Elongation of the cephalothorax.

Castianeirinae - The cephalothorax has undergone moderate elongation in the genera *Castianeira*, *Nucastia*, *Poecilipta*, *Merenius*, and the species *Corinnomma semiglabrum*, *Castianeirodes natalensis*, *Aetius nocturnus*, and *Coenoptychus tropicalis*. The most extreme elongation was limited to *Myrmecium bifasciatum*, *Serendib suthepica*, *Apochinomma formicaeforme*, and one of the *Corinnomma* species.

Myrmarachnini - The elongation of the cephalothorax was at a moderate level in the ancestors of this tribe and improved to high levels of elongation in nearly all descendants. There were however five instances where the elongation was greatly reduced (e.g., *Damoetas* spp.) or even completely lost (e.g., *Myrmarachne sensu lato* CAS103), which has a unique shorter and wider cephalothorax habitus relative to other species in this group.

3) Elongation of the abdomen.

Castianeirinae - Moderate elongation of the abdomen appears to have already been present early in the evolutionary history of Castianeirinae. The most elongated abdomens are found in *Aetius nocturnus*, some *Castianeira* and *Leichhardteus* species, and the genera *Nucastia*, *Poecilipta*, *Kolora*, and *Merenius* while the elongation of the abdomen has been greatly reduced in *Castianeira gertschi*, *Iridonyssus formicans*, *Nyssus avidus*, *Myrmecium bifasciatum*, *Graptartia granulosa*, *Copuetta magna*, *Apochinomma formicaeforme*, and the genus *Coenoptychus*.

Myrmarachnini - Our results suggest that the elongation of the abdomen is quite labile with two clades containing many species with highly elongated abdomens, many instances where the trait has remained moderate, and additionally many instances where the elongation of the abdomen has been reduced or lost.

4) Elongation of the pedicel.

Castianeirinae - The evolution of the elongation of the pedicel is not common among Castianeirinae used in this study. This trait is most highly extended in *Mazax pax*, *Myrmecium bifasciatum*, and *Serendib volans* and moderately extended in *Leptopicia bimaculata*, *Apochinomma formicaeforme*, and some of the *Poecilipta* species.

Myrmarachnini - The extreme elongation of the pedicel is restricted to two clades, a China/Singaporean clade, and a Malagasy clade. Slight elongations of the pedicel occur infrequently in this group with the remaining individuals in the tree possessing no elongation to the pedicel.

5) Illusion by coloration.

Castianeirinae - The appearance of only one aspect of illusion by coloration (i.e., white patches or bands of white on the abdomen) has evolved in only a few taxa used in this phylogeny including *Mazax pax*, *Apochinomma formicaeforme*, *Aetius nocturnus*, *Coenoptychus mutillicus*, *Graptartia granulosa*, *Leptopicia bimaculata*, *Nyssus semifuscus*, *Nyssus luteofinis*, some of the *Castianeira* species and many of the *Poecilipta* species.

Myrmarachnini - The occurrence of illusion by coloration is mostly found in two clades (an African clade and a Singaporean clade) with several scattered cases throughout the tree. There are many instances where there is only one aspect of illusion by coloration and several situations where there is an absence of illusion by coloration.

6) Constrictions of the cephalothorax (dorsal and lateral).

Summary - In Castianeirinae, the lateral constrictions of the cephalothorax have only evolved in three lineages, most notably *Myrmecium* and *Serendib*. The lateral constrictions of the cephalothorax were closely tied with the

evolution of the dorsal constriction in Myrmarachnini and two of the Castianeirinae, *Myrmecium* and *Serendib*. Lateral cephalothorax constriction evolved early in the evolutionary history of the *Myrmarachne sensu lato* group, has been enhanced (greater constriction) in several clades, particularly in individuals collected from Singapore, but has been lost in groups from Malaysia and Madagascar. However, in the Malagasy clade constrictions have subsequently re-evolved.

Castianeirinae - The dorsal constriction of the cephalothorax is extremely rare in Castianeirinae sampled in this study, only appearing in *Myrmecium bifasciatum*. The lateral constriction to the cephalothorax appears in three species used in this study. This constriction is most extreme in *Myrmecium bifasciatum*, moderate in *Serendib suthepica*, and very slight in *Apochinomma formicaeforme*.

Myrmarachnini - More extreme dorsal and lateral constrictions of the cephalothorax are mainly restricted to three clades in Myrmarachnini. The moderate constriction of the cephalothorax is scattered throughout the tree amongst clades of slight constrictions or complete absence of constrictions to the cephalothorax.

7) Constrictions of the abdomen (dorsal and lateral).

Summary - In Castianeirinae, abdominal constrictions evolved only in a few non-related lineages, with dorsal and lateral abdominal constrictions evolving independently, except for a *Castianeira sp*. from South Africa. In Myrmarachnini, abdominal constrictions were more dynamic and scattered across separated lineages compared to cephalothorax constrictions with dorsal and lateral constrictions of the abdomen correlated.

Castianeirinae - The dorsal constriction to the abdomen was found to be most exaggerated in one *Castianeira* species, moderate in *Aetius nocturnus*, and slight in *Mazax pax* and *Poecilipta kohouti*. This trait is absent from all other species used. Few species have evolved lateral constrictions to the abdomen with *Mazax pax*, *Aetius nocturnus*, one of the *Corinnomma* species and a few *Castianeira* and *Poecilipta* species possessing this trait.

Myrmarachnini - The lateral and dorsal constrictions to the abdomen are far less common than the constrictions to the cephalothorax with the more extreme forms appearing mostly in one clade with several phylogenetically distant instances throughout the phylogeny. Moderate constrictions, slight constrictions, and no constrictions of the abdomen are also sporadically distributed throughout the tree.

REFERENCES

Prószyński, J. 2016. Delimitation and description of 19 new genera, a subgenus and a species of Salticidae (Araneae) of the world. Ecol. Montenegrina 7:4–32. 10.37828/em.2016.7.1.
