# Supplementary material for: Batesian Mimicry Converges toward Inaccuracy in Myrmecomorphic Spiders
Source: Syst Biol. 2025 May 19;74(6):967–84. doi: 10.1093/sysbio/syaf037 (PMC12712336; doi:10.1093/sysbio/syaf037)
Supplement: syaf037_Supplemental_Files [file syaf037_supplemental_files.zip › Figure S2.pdf]

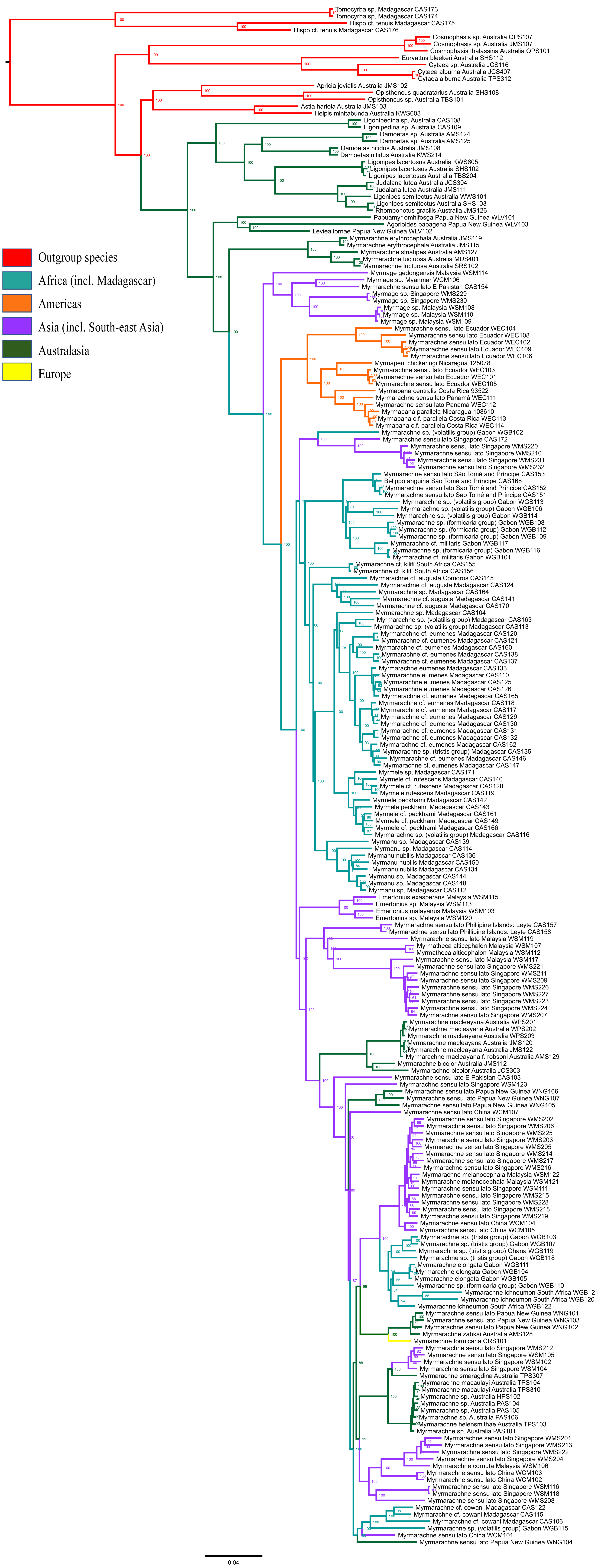

Fig. S2. Maximum likelihood (ML) phylogeny of the salticid tribe, Myrmarachnini, reconstructed using ultraconserved elements (UCEs) (75% occupancy matrix). Inner node values show branch support, estimated with Ultrafast Bootstrap Approximation using 1000 replicates. Branches are coloured indicating the outgroup samples and geographic locations of collection for ingroup (Myrmarachnini) samples. See Legend in figure for geographic information.
