# Supplementary material for: Batesian Mimicry Converges toward Inaccuracy in Myrmecomorphic Spiders
Source: Syst Biol. 2025 May 19;74(6):967–84. doi: 10.1093/sysbio/syaf037 (PMC12712336; doi:10.1093/sysbio/syaf037)
Supplement: syaf037_Supplemental_Files [file syaf037_supplemental_files.zip › Figure S4.pdf]

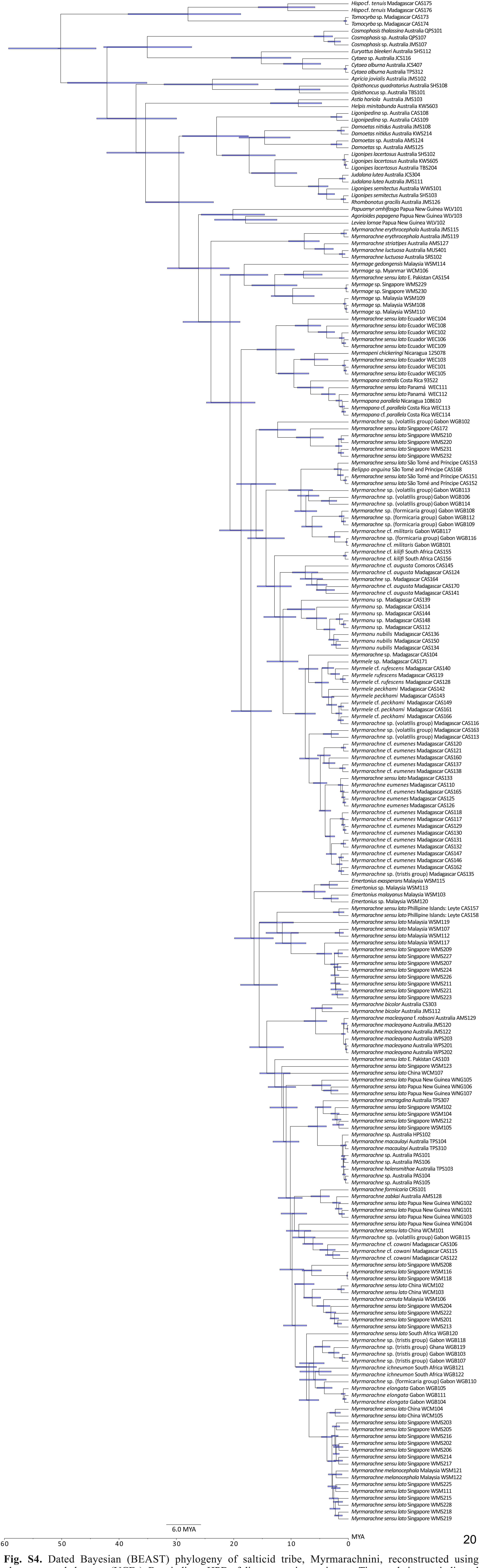

Fig. S4. Dated Bayesian (BEAST) phylogeny of salticid tribe, Myrmarachnini, reconstructed using ultraconserved elements (UCEs). Bars indicate HPD of divergence time estimates. Time scale in mya indicated below.
