# Supplementary material for: Batesian Mimicry Converges toward Inaccuracy in Myrmecomorphic Spiders
Source: Syst Biol. 2025 May 19;74(6):967–84. doi: 10.1093/sysbio/syaf037 (PMC12712336; doi:10.1093/sysbio/syaf037)
Supplement: syaf037_Supplemental_Files [file syaf037_supplemental_files.zip › Figure S5.pdf]

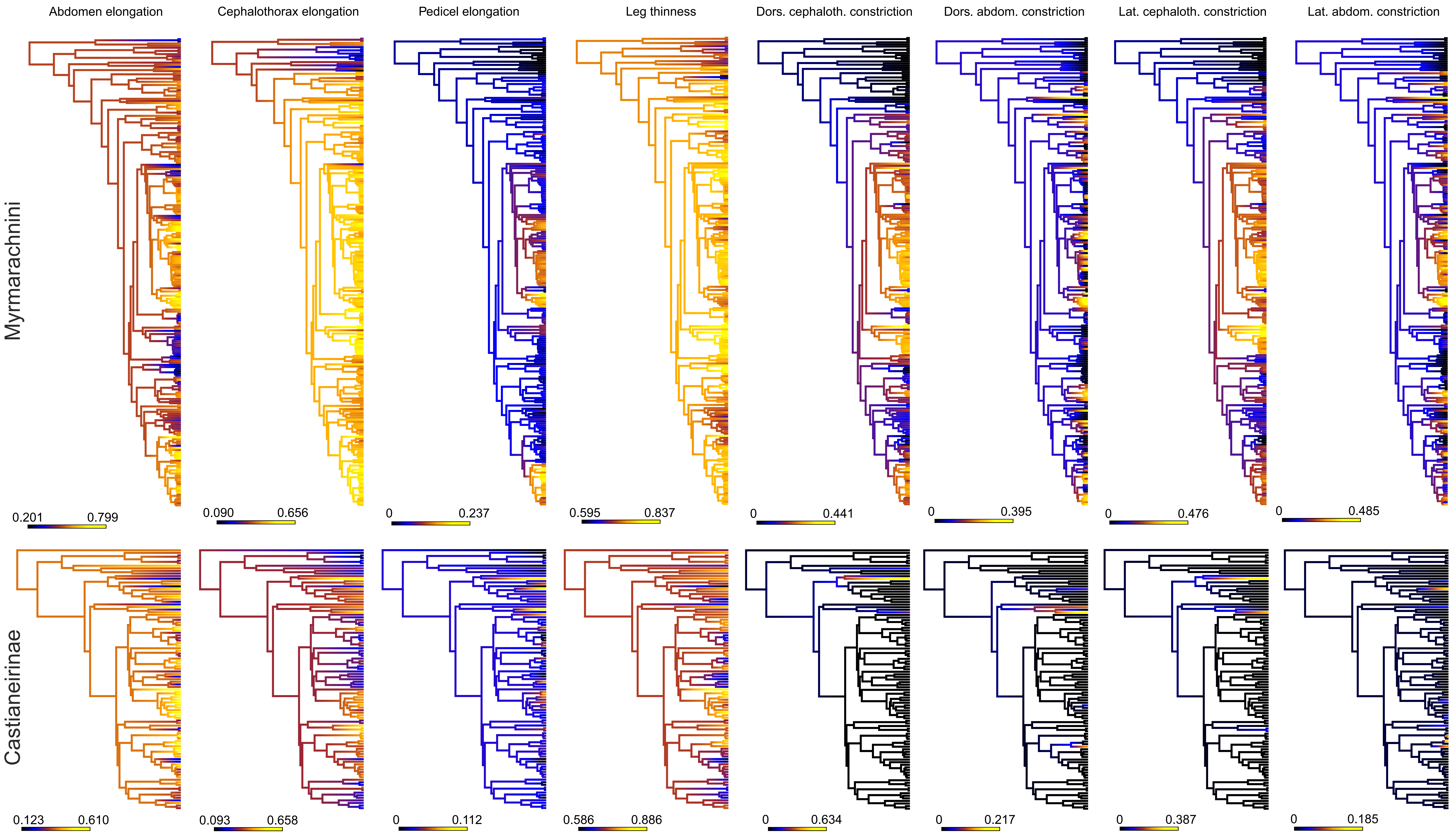

Fig. S5. Phylogenetic ACE plots of eight traits contributing to myrmecomorphy in Myrmarachnini (top row) and Castianeirinae (bottom row). Trait estimates at nodes were based on the best fit of three alternative models (BM, OU and EB).
