# Supplementary material for: Batesian Mimicry Converges toward Inaccuracy in Myrmecomorphic Spiders
Source: Syst Biol. 2025 May 19;74(6):967–84. doi: 10.1093/sysbio/syaf037 (PMC12712336; doi:10.1093/sysbio/syaf037)
Supplement: syaf037_Supplemental_Files [file syaf037_supplemental_files.zip › Table S1.pdf]

**Table S1.** Traits quantified to determine myrmecomorphy, after Kelly et al. (2021).

| *Characteristic* | *Quantification formula* | *Note* |
|---|---|---|
| ***1**: Thin legs* | 1 – width leg III femur / length leg III femur | - |
| ***2**: Elongation of cephalothorax* | 1 – cephalothorax width / cephalothorax length | - |
| ***3**: Elongation of abdomen* | 1 – abdomen width / abdomen length | - |
| ***4**: Elongation of pedicel* | pedicel length / total body length | - |
| ***5**: Constriction of the cephalothorax* | 1 – cephalothorax width at point of constriction / cephalothorax width at widest point | in dorsal view |
| ***6**: Constriction of the cephalothorax* | 1 – cephalothorax height at point of constriction / cephalothorax height at highest point | in lateral view |
| ***7**: Constriction of the abdomen* | 1 – abdomen width at point of constriction / abdomen width at widest point | in dorsal view |
| ***8**: Constriction of the abdomen* | 1 – abdomen height at point of constriction / abdomen height at highest point | in lateral view |
| ***9**: Illusion by coloration* | i) transverse band or stripe of lightly colored setae on the cephalothorax creating the illusion of a separation into a head and thorax | scored based on the presence of the illusion by coloration traits i-iii (i.e., single trait = 0.334, two traits = 0.667, and all three traits = 1.0) |

| | |
|---|---|
| | ii) transverse band or stripe of lightly colored setae on the abdomen creating the illusion of a separation into a petiole (or postpetiole) and abdomen<br><br>iii) darkening of the area surrounding the posterior lateral eye creating the illusion of only two large compound eyes |
| ***Overall mimic accuracy*** | **(A + B + C + D + E + F + G + H + I) / 9** |
