# Supplementary figures and images for: Batesian Mimicry Converges toward Inaccuracy in Myrmecomorphic Spiders
Source: Syst Biol. 2025 May 19;74(6):967–84. doi: 10.1093/sysbio/syaf037 (PMC12712336; doi:10.1093/sysbio/syaf037)

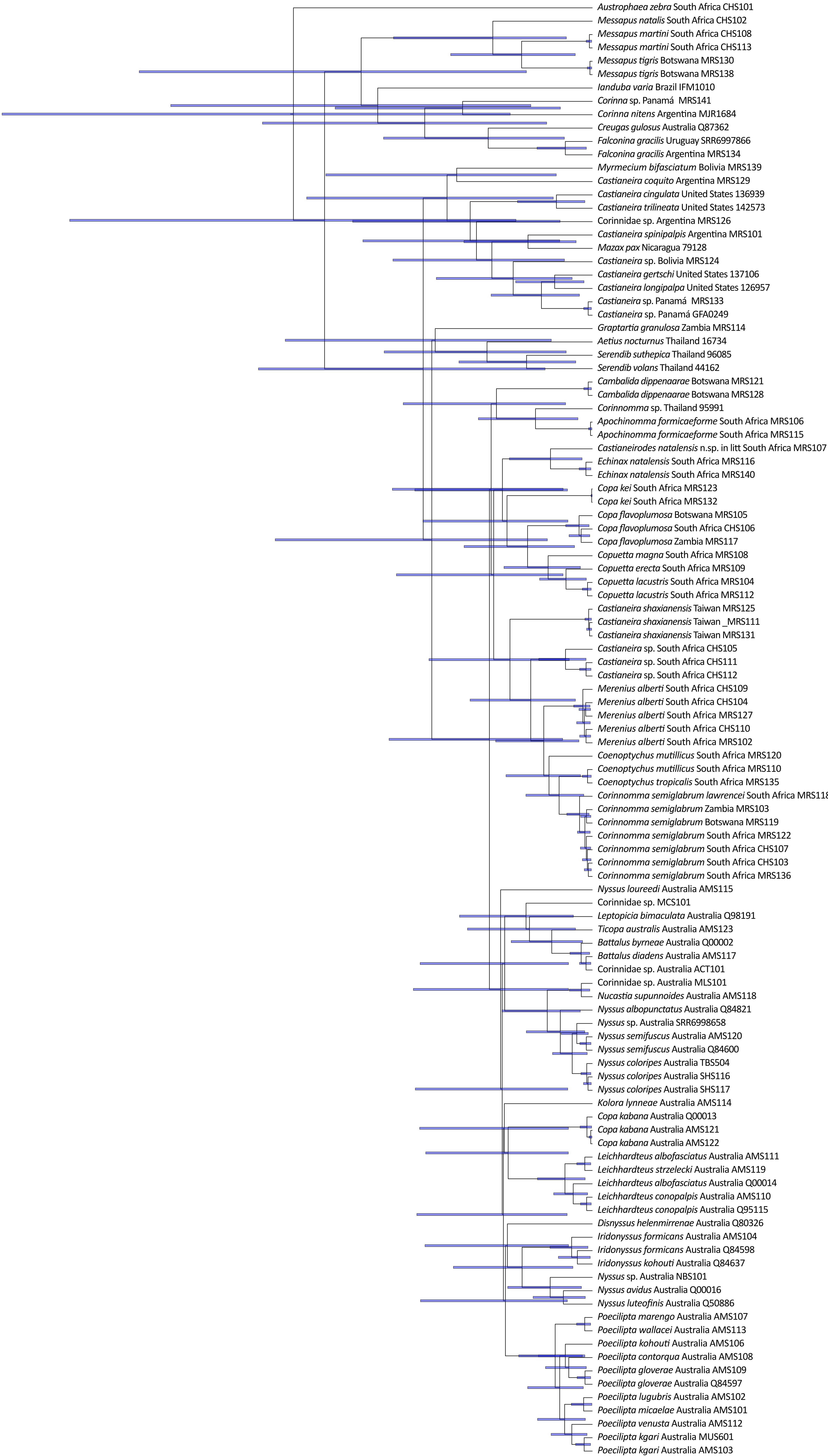

Supplement: syaf037_Supplemental_Files [file syaf037_supplemental_files.zip › Figure S3.pdf]
